# Biomedical Implants with Charge‐Transfer Monitoring and Regulating Abilities

**DOI:** 10.1002/advs.202004393

**Published:** 2021-06-24

**Authors:** Donghui Wang, Ji Tan, Hongqin Zhu, Yongfeng Mei, Xuanyong Liu

**Affiliations:** ^1^ State Key Laboratory of High Performance Ceramics and Superfine Microstructure Shanghai Institutes of Ceramics Chinese Academy of Sciences Shanghai 200050 China; ^2^ School of Materials Science and Engineering Hebei University of Technology Tianjin 300130 China; ^3^ Department of Materials Science Fudan University Shanghai 200433 China; ^4^ School of Chemistry and Materials Science Hangzhou Institute for Advanced Study University of Chinese Academy of Sciences Hangzhou 310024 China

**Keywords:** bioelectronics, cell behavior, charge transfer, electron, biomedical implant, ion

## Abstract

Transmembrane charge (ion/electron) transfer is essential for maintaining cellular homeostasis and is involved in many biological processes, from protein synthesis to embryonic development in organisms. Designing implant devices that can detect or regulate cellular transmembrane charge transfer is expected to sense and modulate the behaviors of host cells and tissues. Thus, charge transfer can be regarded as a bridge connecting living systems and human‐made implantable devices. This review describes the mode and mechanism of charge transfer between organisms and nonliving materials, and summarizes the strategies to endow implants with charge‐transfer regulating or monitoring abilities. Furthermore, three major charge‐transfer controlling systems, including wired, self‐activated, and stimuli‐responsive biomedical implants, as well as the design principles and pivotal materials are systematically elaborated. The clinical challenges and the prospects for future development of these implant devices are also discussed.

## Graphical abstract

Transmembrane transfer of ion and electron (namely, charge transfer) is crucial for cells to interact with the external environment. Implants with charge‐transfer‐controlling abilities can realize real‐time detection and modulation of cell/tissue behavior. This review discusses the mechanism of charge transfer between organisms and implants, and summarizes the recent progresses and perspective of implants with charge‐transfer monitoring or regulating abilities.

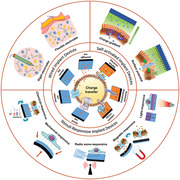

## 1 Introduction

Cell is the basic unit for all living organisms. The cell membrane acts as a barrier to prevent the free entry of extracellular substances into the cell, which ensures the relative stability of the intracellular environment.^[^ ^1^ ^]^ Nevertheless, to maintain a sequence of biochemical reactions inside the cells, cells must incessantly transfer matter and energy with the surrounding microenvironment through cell membranes (i.e., transmembrane transfer).^[^ ^2^ ^]^ Transmembrane transfer of ions and electrons (namely, charge transfer) is the main pathway for cells to interact with the external environment, which plays an important role in cell signaling, cellular metabolism, and the regulation of gene and protein expression.^[^ ^3^ ^]^ In‐depth understanding of how transmembrane charge transfer works and designing biomedical implant materials that can monitor and regulate cellular charge transfer is of significance to manipulate cell behaviors toward tissue repair.^[^ ^4^ ^]^

**Figure 1:**
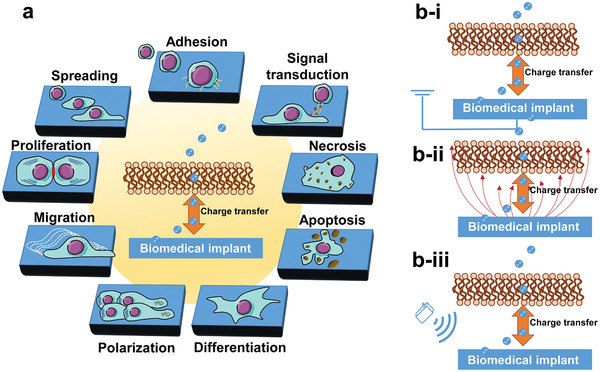
a) Charge transfer drives a series of cellular behaviors, including adhesion, spreading, proliferation, migration, polarization, differentiation, apoptosis, necrosis, and signal transduction. b) Major designs of biomedical implants with charge‐transfer monitoring and regulating abilities: b‐i) wired implant, b‐ii) self‐activated implant, and b‐iii) stimuli‐responsive implant.

Generally, the movement of electrons and ions across the cell membrane creates an electrochemical gradient and affects the overall membrane potential.^[^ ^5^ ^]^ The electrochemical gradient powers the selective transport of ions and molecules, cellular motility, synthesis of adenosine triphosphate (ATP), and redox balance.^[^ ^6^ ^]^ Hence, numerous critical biological processes, including photosynthesis,^[^ ^7^ ^]^ respiration,^[^ ^8^ ^]^ and signal transduction,^[^ ^9^ ^]^ are driven by charge flow across the cell membrane. Besides, transmembrane charge transfer is proved to be able to regulate various cellular behaviors including cell proliferation, migration, differentiation, and apoptosis (**Figure** **1**a).^[^ ^10^ ^]^ In addition, it is well established that the transmembrane charge flow is crucial in important biological processes after cells organize into tissues, including signal transmission in neural and muscle,^[^ ^11^ ^]^ embryogenesis, wound healing, tissue repair, and remodeling as well as normal growth of organisms.^[^ ^10^, ^12^ ^]^ Therefore, transmembrane charge transfer forms an epigenetic pathway that could be potentially used as a tool to analyze and control biological behavior. Designing implant devices that can sense or modulate transmembrane charge transfer have the capacity to achieve two goals: 1) monitoring cellular activities and communicating with the host cells or tissues, and 2) manipulating cellular behavior, and realizing specific physiological functions.^[^ ^4^ ^]^ Thus, synthetic bioelectronic interfaces that seamlessly exchange information and energy across the boundary between living and human‐made systems can be fabricated.^[^ ^3^ ^]^

Currently, researchers have established a large number of advanced implants that sense or affect the transmembrane charge‐transfer process of around cells or tissues. Unlike traditional implant which can only provide passive physical supports, these advanced forms of biomedical implants with charge‐transfer regulating abilities enable cross‐talking with the host tissues,^[^ ^13^ ^]^ and they realize closed‐loop health monitoring and advanced feedback therapy such as in situ active programmed stimulation in the brain,^[^ ^14^ ^]^ cochlear,^[^ ^15^ ^]^ or retinal prostheses.^[^ ^16^ ^]^ To regulate the transmembrane transport of ions and electrons, electrical sources are usually incorporated into the devices. Based on the types of power suppliers, there are three types of implantable devices with charge‐transfer controlling abilities: 1) wired implants, which can monitor and regulate charge flow through a wired external circuit attached to the implants (Figure 1b ‐i);^[^ ^17^ ^]^ 2) self‐activated implants, which can induce charge transfer without any external energy input (Figure 1b ‐ii);^[^ ^18^ ^]^ 3) stimuli‐responsive implants, which can regulate charge transfer by coupling with external stimuli such as light, ultrasound, and magnetic field (Figure 1b ‐iii).^[^ ^19^ ^]^ Some charge‐transfer controlling devices, specifically those powered by external circuses such as heart pacemakers and neurological probes, have already available in the clinic.^[^ ^20^ ^]^ However, self‐activated and stimuli‐responsive biomedical implants that can sense or regulate charge transfer are rarely commercially available. Nevertheless, various de novo wireless implants have been designed, and studies on their clinical potential have been widely investigated by in vivo animal experiments.^[^ ^21^ ^]^

In this review, we describe basic design principles, de novo materials, and the working mechanism of implants with charge‐transfer monitoring or modulating abilities. The transmembrane charge‐transfer mechanisms are summarized first. Subsequently, we introduce recent progress of implant devices with charge‐transfer controlling abilities energized via various sources: wired, self‐activated, and stimuli‐responsive (Figure 1b). Pivotal materials applied in different types of charge‐transfer controlling implants are described. In addition, the challenges and future development trends of each type of implant with charge‐transfer monitoring or regulating abilities are clarified.

## 2 Pathways of Transmembrane Charge Transfer

Transmembrane charge transfer is considered to be essential for cell development and tissue homeostasis. Various critical biological processes such as respiration and transport of nutrients are motivated by the flow of electrons and ions across the membrane of living cells. As a representative example, the energy generation in bacteria is a charge‐transfer process in essence. ATP is the fundamental energy currency for all lives, which is involved in most chemical reactions within the cells. The ATP synthetic process of bacteria is based on two linked charge‐transfer stages:^[^ ^22^, ^169^ ^]^ 1) bacteria pump protons out of cells with the action of respiratory electron transport chain embedded in the bacterial membrane to establish a transmembrane electrochemical proton gradient; 2) this gradient pulls extracellular protons flow back into bacteria through ATP synthase to produce ATP. The charge‐transfer process is also reported to affect tissue function. It is widely known that muscle, glandular tissue, and nerve systems transmit signals/impulses by taking advantage of the charge‐transfer process.^[^ ^11^ ^]^ In addition, a series of biological processes including wound healing, embryogenesis, and tissue remolding are related with charge‐transfer processes .^[^ ^10^, ^23^ ^]^ As demonstrated in a landmark research conducted by Borgens,^[^ ^24^ ^]^ living bone tissue drives ionic transfer through itself and to injured sites, which results in an electric current. Such “injured current” consists of a persistent current and an intense decaying current depending on bone deformation. The persistent current is mainly driven by the transfer of Cl^−^, and to a minor extent by Na^+^, Mg^2+^, and Ca^2+^. Such endogenous charge transfer is involved in bone repair, remodeling, and growth; and regulating ionic flow by electrical stimulating has been successful in treating chronic nonunion in damaged bone tissue.^[^ ^24^ ^]^

Ions and electrons can pass across the cell membrane through passive or active ways. Most ions can only be transported by diverse

membrane proteins, including porins, ionophores, ion channels, and ion pumps, while a few ions (Cs^+^, I^−^, and ClO_4_^−^) can move across the cell membrane through free diffusion.^[^ ^1^, ^2^ ^]^ In contrast, transmembrane electron transfer is hardly achieved without the help of membrane proteins. Electron‐transfer proteins carry electrons from donors on one side of the lipid bilayer to the acceptors on the other side. These proteins usually contain redox centers for electron delivery. Additionally, molecular recognition elements are found in electron transfer proteins, which enable exquisitely selective transport from specific electron donors to specific acceptors.^[^ ^3^ ^]^ In general, cellular transmembrane charge transfer is strictly controlled by these proteins. Disruption of the transmembrane transport of electrons or ions will result in various physiological disorders. For instance, abnormal transportation of chloride or sodium ions leads to myotonia;^[^ ^25^ ^]^ excess extracellular transport of H ion gives rise to hyperacidity.^[^ ^26^ ^]^ Notably, uncontrolled transmembrane transfer of H ions might result in death.^[^ ^27^ ^]^ Therefore, it is essential to understand how transmembrane charge transfer occurs in nature.

### 2.1 Ion Transfer

**Figure 2:**
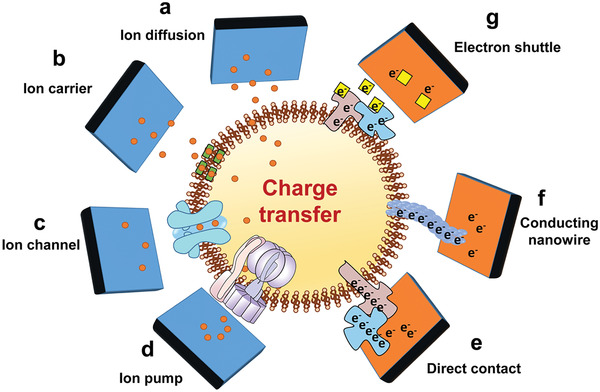
Charge transfer between implants and cells through a) diffusion, b) ion carrier, c) ion channel, d) ion pump, e) direct contact, f) conducting nanowires, and g) electron shuttle.

In general, there are two modes for ions to transport across the cell membrane: passive transport and active transport.^[^ ^28^ ^]^ During passive transport, ions will transport along the chemical potential gradient, and no energy is consumed in the whole transfer process. Passive transport can be further divided into free diffusion and facilitated diffusion. Main difference between the two is that facilitated diffusion requires presence of specific integral membrane proteins, which are known as ionophores or ion carriers, to help transport ions across the cell membrane, while free diffusion does not require carriers. Active transport is a totally different pathway, which is characterized by saturability and selectivity, and requires energy expenditure. Active transport is mediated by membrane proteins known as ion channels or ion pumps. They can realize ion transport against the chemical potential gradient. As shown in **Figure** **2**, ions can be transported between cells and materials in both passive and active manners with the aid of different types of membrane proteins.

#### 2.1.1 Passive Transport

Free diffusion is the simplest ion transfer process between cells and material, and it is the only ion transfer mode that can proceed without the facility of any kinds of membrane proteins. In a typical free diffusion process, ions diffuse along the concentration gradient from the high concentration to the low concentration side (Figure 2a). The free diffusion rate of ions is based upon their sizes, charges, lipid solubility, and transmembrane concentration gradients.^[^ ^29^ ^]^ The diffusion flux (*j*_s_) of different ions can be calculated based on the following equation

(1) $$j_{s}\;=\frac{\Delta cBD}{L}\;$$

where Δ*c* is the transmembrane concentration gradient, *B* is the distribution coefficient, which can be obtained by calculating the ratio of the ion concentration in the aqueous phase to the lipid layer, *D* is the diffusion coefficient of ions in the lipid layer, and *L* is the thickness of the lipid layer.^[^ ^30^ ^]^ Owing to the poor lipid solubility of hydrophilic ions such as Na^+^, K^+^, and Cl^−^, their *D*s are very small, thus the cell membrane is a high‐energy barrier for hydrophilic ions. A protein‐free bilayer of phosphatidylcholine in 0.1 m NaCl solution has a specific conductance of 1.3 nS cm^−2^,^[^ ^31^ ^]^ corresponding to the conductance of an excellent insulator. This large barrier for hydrophilic ions is crucial for the functioning of the cell membrane. This is because it allows the selective passage of certain types of ions and enables the cell membrane to regulate the ionic permeability via chemical or electrical means. In contrast, it is easier for ions with higher hydrophobicity, such as Cs^+^, I^−^, and ClO_4_^−^, to pass through the lipid layer.^[^ ^32^ ^]^ Hydrophobic ions tend to distribute into the bilayer lipid membrane (BLM), attracting counter hydrophilic ions into the BLM to maintain the electroneutrality within the membrane.^[^ ^33^ ^]^ Hence, the diffusion rate of the hydrophilic ions can be enhanced by the hydrophobic ions with opposite charges, even if the existing hydrophobic ions are slightly dilute (e.g., 10^−6^ m).^[^ ^34^ ^]^ However, most ions in the biological systems are hydrophilic, and the *D* of these ions in the cell membrane is slightly small, far less than that of water molecules. Therefore, once the cells are exposed to a microenvironment with a very high or low ion concentration, high osmotic pressure will cause plenty of water molecules to diffuse through the cell membrane at the express speed, resulting in the death of the cell because of swelling or dehydration. For instance, the degradation products of biomedical Mg alloys are not toxic, but cells hardly survive on their surface, which can be ascribed to the high osmotic pressure induced by the rapid and massive release of Mg^2+^ ions from the Mg alloys.^[^ ^35^ ^]^

Facilitated diffusion is another type of passive transport mediated by membrane proteins known as ionophores, which are molecules that bind specific ions and then transport them in a bound form through the membrane (Figure 2b). Various compounds that function as mobile ion carriers, such as macrotetrolides (monactin) and macrocyclic compounds (valinomycin, enniatin B), have been known so far.^[^ ^36^ ^]^ In most cases, the hydrophilic ions bind with the ion carrier to form a complex with a strong hydrophobic exterior, which overcomes the hindrance to move the hydrophilic ions from the aqueous phase into the apolar interior of the cell membrane.

#### 2.1.2 Active Transport

Active transport of ions requires the assistance of ion channels or ion pumps on the cell membrane. Ion channels formed by transmembrane proteins are multifarious hydrophilic pores with ion selectivity, and they can be switched on and off by various physicochemical stimulations (Figure 2c). According to the signal controlling their on–off states, ion channels can be divided into ligand gating, voltage gating, and mechanosensitive channels.^[^ ^37^ ^]^ The ligand gating channel is regulated by extracellular ligands, intracellular second messengers, metabolites, protein interactions, or phosphorylation. The voltage gating channel is sensitive to membrane potential, whereas the mechanosensitive channel is regulated by the stress of the cell membrane. Once the ion channel is switched on, ions can diffuse along the concentration gradient freely, and no extra energy is expended during the process. Notably, ion channels play an important role in the transmission of nerve signals.^[^ ^38^ ^]^ With regard to a neuron in resting state, only K^+^ leak channels are opened and the opening of other ion channels is strictly regulated. Therefore, K^+^ ions transport across the membrane in an unregulated manner, and the overall membrane potential is close to the resting potential for K^+^ ions, which is about −70 mV. Once stimuli are applied to the neuron, ligand‐gated Na^+^ channels open, and positive charged Na^+^ ions enter into cells making the membrane potential gradually increase to −40 mV. Then, voltage‐gated Na^+^ channels are activated, Na^+^ ions rush into the neuron, resulting a rapid increase of the membrane potential. When the membrane potential reaches 40 mV, the voltage‐gated Na^+^ channels become inactivated, while the voltage‐gated K^+^ channels open slowly, K^+^ ions rush out the cell. The repolarization of the cell lowers the membrane potential, leads to closure of voltage‐gated Na^+^ and K^+^ channels; thus, ion concentration gradually returns to resting level. The transmembrane ion transfer induces membrane potential alteration propagates along the neuronal axon; thus, neural signal transmission is realized. Recent research showed that ion channels are also essential in regulating bacteria interactions through transportation of K^+^,^[^ ^39^ ^]^ Mg^2+^,^[^ ^40^ ^]^ and Ca^2+^.^[^ ^41^ ^]^ Prindle et al.^[^ ^39^ ^]^ found that ion channels conduct long‐range electrical signals within bacterial biofilm communities through spatially propagating waves of K^+^. A metabolic trigger induces the opening of the K^+^ channel, and the release of intracellular potassium, which in turn depolarizes neighboring cells. There is a link between membrane potential and metabolic activity, so cells can rapidly communicate their metabolic state via the K^+^ channel‐mediated electrical signal. The depolarization wave that is triggered by metabolically stressed interior cells would limit the nutrients‐taking‐up abilities of cells in the biofilm periphery; thus, allowing interior cells more access to the nutrients. Other kinds of ion channels are also reported to regulate bacterial behaviors. Research conducted by Lee et al.^[^ ^40^ ^]^ indicated that ion channel‐mediated transmembrane Mg^2+^ flux, directly affected ribosome function and increased the resilience of bacteria to ribosome‐targeting antibiotics.

Unlike ion carriers and channels, which only allow ions to be transported along chemical potential gradients, ion pumps enable ions to be actively transported across the cell membrane against the electrochemical potential gradient with an adequate energy supply (usually from the hydrolysis of ATP).^[^ ^42^ ^]^ Moreover, ion pumps can run in reverse to transfer ions from the high to the low concentration regions, accompanied by the synthesis of ATP (Figure 2d). Therefore, the reverse‐running ion pump is actually an ATP synthase, which can convert potential energy stored in the transmembrane ion gradient into chemical energy stored in the ATP. In fact, this is how cells obtain usable energy, and thus the normal running of ion pumps is crucial to maintain cell function.

### 2.2 Electron Transfer

Studies have increasingly established that some microorganisms can transfer electrons outside the cells, this is known as extracellular electron transfer (EET).^[^ ^43^ ^]^ Nevertheless, it is still under debate how the endogenously produced electrons pass to the extracellular material.^[^ ^44^ ^]^ Currently, it is clear that the EET of mineral‐respiring bacteria depends on a haem‐based electron transfer mechanism. The electrons produced by intracellular oxidation of organic substances are transferred to the outer membrane through the redox effect of various cytochrome C in the inner membrane, periplasmic space, and outer membrane.^[^ ^45^ ^]^ This series of cytochromes form a transmembrane electron transport chain that crosses the nonconductive cell membrane.^[^ ^43^, ^46^ ^]^ There are various ways for transporting electrons from the cytochrome C on the cell membrane to extracellular materials, including direct transfer, nanowire, and shuttle‐mediated electron transport. 1) Direct transfer (Figure 2e): electrons can be directly transferred from the cytochrome C in the outer membrane to the materials when the extracellular material contacts or is very close to the outer membrane of the microorganism.^[^ ^47^ ^]^ 2) Nanowire (Figure 2f): nanowire is conductive pili or flagellate structure synthesized by cells.^[^ ^48^ ^]^ It connects the bacteria to the electron acceptor physically and electrically, thereby mediating long‐distance electron transport, when the bacteria cannot directly contact the electron acceptor.^[^ ^49^ ^]^ These nanowires were previously misidentified as type IV pili. Recently, a cryoelectron microscopy structure with a resolution of 3.7 Å of the conducting nanowires that established the molecular basis for electronic conductivity in the nanowires was obtained. This revealed that the nanowires were assembled by micrometer‐long polymerization of the hexaheme cytochrome OmcS, with hemes packed within 3.5–6 Å.^[^ ^50^ ^]^ 3) Electron shuttle (ES)‐mediated electron transport (Figure 2g): ESs, also known as redox mediators, are special electron carriers that can reversibly participate in redox reactions with the ability to accept and give electrons.^[^ ^51^ ^]^ ESs are classified as endogenous and exogenous, which can mediate both the output of intracellular electrons and the input of extracellular electrons. Endogenous ESs are electron tranport substances produced by microorganisms and secreted outside, such as flavins, melanin, etc.^[^ ^52^ ^]^ Exogenous ESs include a variety of redox substances that are either naturally, or artificially synthesized such as humus and quinones substance, biochar, Fe_3_O_4_ nanoparticle, etc.^[^ ^53^ ^]^ Both endogenous and exogenous ESs have the ability to receive and give electrons repeatedly, and they can act as a bridge to transport electrons between cells and materials.^[^ ^54^ ^]^ For instance, *Shewanella* sp. secrete redox‐active flavin compounds, which are able to transfer electrons between the cell surface and substrate in a cyclic fashion.^[^ ^55^ ^]^

Except for mineral‐respiring bacteria, the existence and mechanistic basis of other EETs is still largely unsuspected. Recently, a distinctive EET mechanism was proposed. The researchers established that the food‐borne pathogen uses a novel flavin‐based EET mechanism to deliver electrons to iron or an electrode, instead of the haem‐based electron transfer mechanisms illustrated above. This mechanism has no elaborate multihaem apparatus, partly by taking advantage of the single‐membrane architecture of the gram‐positive cell, and it is characterized by significantly fewer electron transfer steps than comparable systems in mineral‐respiring gram‐negative bacteria.^[^ ^56^ ^]^ It can be observed that the mechanism of charge transfer between materials and organisms is the hot field in biological, material, and physical research. New findings in this field are springing up, which will provide new insights into the design of implant materials with charge monitoring and regulating abilities.

## 3 Strategies to Monitor and Regulate the Transmembrane Charge Transfer

### 3.1 Charge‐Transfer Monitoring

The transmembrane charge transfer alters the charge distribution across the cell membrane, thus changing the electrical potential of cell membrane. Therefore, the membrane potential alteration (so‐called bioelectrical signals) is the reflection of transmembrane charge transfer and can be detected by recording the voltage or current at the interface of implant/organism. Tissue‐level charge‐transfer detecting has been commercially achieved by electroencephalography (EEG),^[^ ^57^ ^]^ positron emission tomography (PET),^[^ ^58^ ^]^ and functional magnetic resonance imaging (fMRI).^[^ ^59^ ^]^ However, the charge‐transfer recording in cell scale is still under development and puts forward a high demand for detecting precision.

The bioelectrical signals induced by charge transfer in cell scale can be recorded from either inside or outside the cells. Monitoring electrical signals from inside of cells can improve signal strength and quality.^[^ ^60^ ^]^ The gold standard for transmembrane charge‐transfer detection, patch‐clamp recording, is an intracellular recording technique. In a conventional patch‐clamp configuration, a glass pipette containing electrolyte solution is tightly sealed onto the cell membrane, achieving “gigaseals,” and thus isolates a membrane patch electrically. Currents fluxing through the channels in this patch hence flow into the pipette and can be recorded by an electrode that is connected to a highly sensitive differential amplifier. The accurate charge‐transfer recording of patch‐clamp requires a high‐resistance seal, the cell membrane must be punctured and allowed to reseal around the electrode,^[^ ^61^ ^]^ and hence the long‐term stability of patch‐clamp is poor. In this section, we mainly focus on the strategies to endow implant devices with bioelectrical signal recording abilities, which is more suitable for the long‐term monitoring of charge transfer in vivo.

#### 3.1.1 Voltage Recording

**Figure 3:**
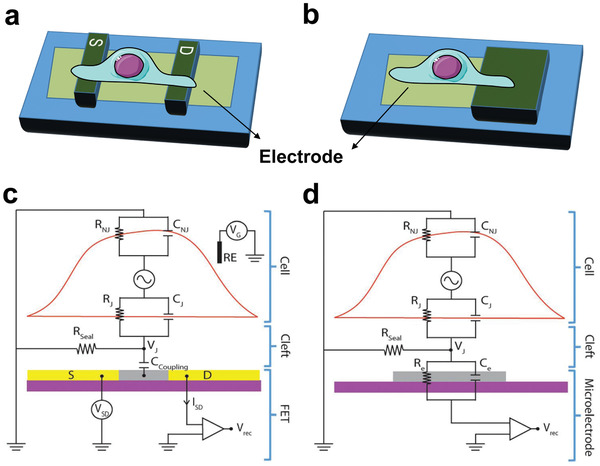
Schematic illustration of implants with charge‐transfer monitoring abilities through a) voltage recording and c) current recording. Electrical equivalent circuit of the b) cell‐electrode and d) cell‐FET interfaces. S and D represent source and drain leads, respectively. *R*_J_, *R*_NJ_, *R*_seal_, and *R*_e_ represent junctional, nonjunctional, seal, and electrode resistances, respectively. *C*_J_, *C*_NJ_, *C*_Coupling_, and *C*_e_ represent junctional, nonjunctional, coupling, and electrode capacitances, respectively. *V*_J_, *V*_SD_, *V*_G_, *V*_rec_, and ISD represent junctional voltage across the cleft, source–drain voltage, gate voltage, recorded voltage, and source–drain current, respectively. RE represents reference electrode. Reproduced with permission.^[^ ^127a^ ^]^ Copyright 2018, The Royal Society of Chemistry.

In order for an implant to detect voltage signals induced by the transmembrane charge transfer, the implant should be connected with an external circuit and served as an electrode (**Figure** **3**a). Transmembrane charge transfer leads to an extracellular potential difference known as junctional voltage (*V*_J_). When the electrode is interfaced with the cells, the *V*_J_ gives rise to a change in the record potential, thus enabling real‐time monitoring of the transmembrane charge transfer (Figure 3b). It is an automatic process that the surface potential of electrodes varied with *V*_J_, so the voltage recording implants are noted as passive devices, and can be easily constructed by connecting the electrodes to a voltmeter. Standard needle or planar electrodes are rigid and large, leading to the poor resolution in voltage recording. In addition, signal attenuation stemmed from the electrode/tissue interface resistance is inevitable in the detection. Therefore, it is a great challenge to sense the charge transfer of a single cell with the voltage recording implant, especially in the complex in vivo environment. The development of micromachining technology has brought numerous opportunities to fabricate novel electrodes for voltage recordings, such as microelectrode array (MEA),^[^ ^62^ ^]^ nanoneedle electrodes, nanomesh electrodes, and patterned metal grids. These advanced types of electrodes have attracted significant attention regarding the monitoring of cell‐level charge transfer, which can realize multisite long‐term detection with submillisecond time resolution.^[^ ^63^ ^]^

#### 3.1.2 Current Recording

Electrodes of the voltage recording implant can be directly used in current recording. Applying a suitable voltage on the electrode, an electric field forms around the implant, and charges will move along the potential gradient to produce an electric current. The magnitude of the current is determined by the applied voltage and charge concentration. Transmembrane charge transfer alters the local charges concentration, and hence affects the recording current. Different from voltage record devices, the voltage needs to be preloaded for current recording implants, and they are referred to as active devices. The loaded voltage will affect the behavior of cells, so the charge transfer of cells may be influenced in the current recording process, and the cellular behavior will be altered accordingly. Some researchers take advantage of this effect to achieve specific physiological functions along with the current recording. For example, Wang et al.^[^ ^64^ ^]^ constructed a gold/zinc oxide (Au@ZnO) layer on the surface of Ti. Charge transfer between the film and bacteria was observed, which was used as a signal to achieve real‐time monitoring of bacteria amount. They established that the current induced by the charge transfer between bacteria and the constructed film and the semilog of bacteria amount had a linear relationship. In addition, the charge transfer between bacteria and materials can destroy the bacterial respiratory chain, thus the detection platform has a highly effective antibacterial ability.

However, in most cases, we do not want to affect the charge‐transfer process in the bioelectrical signal detection, which will result in signal distortion. Scaling down the preloaded voltage can decrease the interference of current recording to the original signal, but can induce lowered detecting resolution. Field‐effect transistors (FETs) are able to transform small voltage signals into large current signals. Replacing traditional electrodes with FET is an effective strategy to lower the preloaded voltage without sacrificing the detecting sensitivity. A conventional FET device consists of a semiconductor substrate (such as p‐type silicon), a source electrode for injecting current, a drain electrode for collecting current, and a gate electrode for controlling the current in the channel between the source and drain electrodes. In general, the source and the drain are made of semiconductors, but their doping type is opposite to that of the substrate (such as n‐type silicon). An insulating oxide layer exists between the gate electrode and the substrate, and the gate electrode is capacitively coupled to the semiconductive channel between the source and drain electrodes. If no voltage is applied at the gate, the FET acts as two back‐to‐back linked PN junctions, thus the current can hardly flow between the source and drain. That is, the transistor is in the off state. When the gate voltage exceeds the threshold, charge carriers are induced in the channel, causing the channel barrier to drop, thus resulting in a significant tunneling current flow. Therefore, the conductance between the source and the drain electrodes can be regulated by the potential applied to the gate electrode. For the FET used to monitor charge transfer in vivo, cells directly contact the channel between the source and drain, acting as the gate (Figure 3c). The gap between the cell and transistor results in a resistance, which is called seal resistance represented by *R*_seal_. The *R*_seal_ can function as an insulating layer between the gate and the channel in a conventional FET device. When the transmembrane charge transfer of cells occurs, the extracellular potential changes, causing the alteration of current between the source and drain electrodes. Thus, the real‐time monitoring of charge transfer between cells and the environment is successfully realized (Figure 3d).^[^ ^65^ ^]^ The development of nano/microfabrication technology provides opportunities to improve the spatial–temporal resolution of FET‐based implants further. Nanosized FET devices, especially nanowire FETs have gained increasing attention in the current signal recording. It is reported that 3D nanowire FETs have realized the charge‐transfer recording of single cells.^[^ ^66^ ^]^ The sensitivity of nanowire FETs is reported to be comparable with that of patch‐clamp electrodes in the charge‐transfer recording of cardiac cells, but the production of nanowire FETs is difficult to scale up. In a recent study, Zhao et al.^[^ ^67^ ^]^ combined shape‐controlled nanowire transfer with spatially defined semiconductor‐to‐metal transformation, and realized scalable fabrication of nanowire FET arrays with controllable tip geometry. The constructed implant allowed recording of up to 100 mV potential alteration stemmed from charge transfer and enabled multiplexed recording from single cells.

### 3.2 Charge‐Transfer Regulating

The transmembrane charge transfer of cells is determined by the following physicochemical factors: 1) the electrochemical potential gradient of the charge (charge tends to move along the chemical potential gradient;^[^ ^1^ ^]^ 2) the activation of charge‐transfer‐related channels.^[^ ^68^ ^]^ In this section, various strategies to endow implant with charge‐transfer regulating abilities will be illustrated.

#### 3.2.1 Regulating the Electrochemical Potential

The electrochemical potential of a substance can be calculated using the following formula

(2) $$\tilde{\mu }=\mu^{0}\;+RT\ln\gamma c+ZF\varphi$$

where ($\tilde{\mu }$) is the electrochemical potential, *μ* ^0^ is the chemical potential of the charge under standard conditions, *R* represents the gas constant, *T* is the temperature, *Z* represents the electric charge of the particle, *γ* represents the activity coefficient, *F* is the Faraday's constant, *c* represents the concentration of charged particles, and *ϕ* is the electric potential. Under certain conditions, *μ* ^0^, *R*, *T*, *Z*, *γ*, and *F* are constants, and only *c* and *ϕ* can be controlled. Therefore, the main approaches to regulate transmembrane charge transport are changing the charge concentration or the electric potential of the implant.

The ion concentration gradient is one of the main driving forces for charge transfer. Through a suitable design, the concentration of specific ions around the implant can be increased or decreased selectively; therefore, the transmembrane ion gradient can be artificially changed, realizing the regulation of ion transfer. Since various ions act as active sites or messenger molecules of enzymes, the cell behavior can be modulated accordingly.^[^ ^69^ ^]^

Modifying the surface potential of the implant is another strategy to regulate the charge transfer, which can be realized by applying an external or internal electric field between the implant and cells.^[^ ^70^ ^]^ Then, the transmembrane charge transfer can be regulated through capacitive or Faradaic charge injection mechanisms.^[^ ^10a^ ^]^ For the capacitive mechanism, there is no direct charge exchange between the implant material and the cells, but only the charge amount on the surface of the implant changes. Thus, the charged surface absorbs some foreign ions in the physiological environment, resulting in the generation of a directional electric field. Thereafter, ions in the environment will migrate along the electric field, which can change the potential of the cell membrane and the concentration gradient of surrounding ions, thus affecting cellular behaviors. For the faradaic charge injection mechanism, direct charge exchange takes place between the implant material and the biological tissue, which will produce an electrical stimulation to cells and thus affecting their behaviors. Generally, when electrical stimulation is applied to cells, the cellular microenvironment and membrane properties undergo a series of complicated changes, inducing relevant changes in cellular behavior.

A large number of studies have shown favorable effects in nerve, muscle, bone, skin restorations, as well as other biological fields by applying electrical stimulation.^[^ ^71^ ^]^ However, the relevant underlying mechanism is unclear, and some studies believe that the regulatory effect may be attributed to the fact that electrical stimulation can change the intracellular concentration of calcium ions.^[^ ^72^ ^]^ The presence of an electric field changes the conformation of calcium channels embedded in the cell membrane and increases the intake of calcium ions.^[^ ^73^ ^]^ Calcium ion is an important messenger, and variation of its intracellular content mediates a series of subsequent changes in cell behavior.^[^ ^74^ ^]^ While some researchers argued that calcium ion channels do not play a role in the electric‐field‐induced changes in cell behavior, many other possible theories on this issue have been proposed. For instance, the electrical stimulus may cause the redistribution of surface receptors on the cell membrane,^[^ ^75^ ^]^ or affect the production of ATP synthesis,^[^ ^76^ ^]^ heat shock proteins,^[^ ^77^ ^]^ reactive oxygen species,^[^ ^78^ ^]^ and lipid rafts.^[^ ^79^ ^]^ Thrivikraman et al.^[^ ^10a^ ^]^ provided a detailed overview of the mechanisms by which electric fields regulate cellular behaviors.

#### 3.2.2 Controlling the Activation of Charge‐Transfer‐Related Proteins

The charge‐transfer‐related proteins are known to respond to numerous cues within the microenvironment such as chemical agents, temperature,^[^ ^80^ ^]^ electrical potential,^[^ ^81^ ^]^ osmolarity, and mechanical stimuli.^[^ ^37^, ^82^ ^]^ Implants that modulate the above factors are expected to control the activation of the charge‐transfer‐related proteins.

Agents that regulate charge channels can be divided into three chemical categories: metal ions, organic small molecules (molecular weight 200–500 Da), and peptides (molecular weight 3–6 kDa). These chemical agents can be loaded onto implants, and regulate the charge transfer via opening or blocking the ion‐conducting pore, or modifying channel gating through binding to the auxiliary subunits. In addition, the introduction of these chemical agents can affect the expression of ion channel proteins, thus affecting the uptake of ions further. In a previous study, Zn‐doped Ti surfaces were fabricated via microarc oxidation (bulk dope) and plasma immersion ion implantation (surface dope). The expression of Zn‐transport‐related proteins was attributed to the Zn doping strategies.^[^ ^83^ ^]^ Although the concentration of Zn ion released from the bulk‐doped sample was relatively high, it promoted the expression of ZnT1, a zinc transporter, which accelerates the removal of Zn ions from the cells. However, the surface‐doped samples did not affect the expression of ZnT1, and in the late culture stage, it promoted the expression of ZIP1, which possessed the ability to transport Zn ions to the cell. Therefore, the surface‐doped samples presented better osteogenic ability than the bulk‐doped samples.

Diverse mechanosensitive charge‐transfer‐related proteins exist on cell membranes across all kingdoms of life forms, including epithelial sodium channel/degenerin (ENaC/DEG)‐superfamily proteins, piezo, two pore‐domain potassium ion channels (K2P/KCNK), transient receptor potential (TRP) superfamily, and transmembrane protein 16/Anoctamin (TMEM16/Ano).^[^ ^37^ ^]^ These ion channels respond to a variety of mechanical stimuli, from osmotic stress to local mechanical deformation. Therefore, implants that impose mechanical tension, stretch, or shear stress on cells are able to regulate the charge transfer. Constructing flexible nanostructures on implant surfaces is an effective strategy to apply mechanical stimuli to cells. When cells attach to the flexible nanostructures, the nanostructures bend and stretch the cell, which results in membrane tension.^[^ ^84^ ^]^ Similarly, membrane tension can be altered by culturing cells on implants with different stiffness.^[^ ^85^ ^]^ It is reported that macrophages cultured on stiff substrates exhibited increased phagocytic capacity and lipopolysaccharide (LPS)‐mediated intracellular calcium influx compared to cells on soft surfaces.^[^ ^86^ ^]^ More advanced types of implants allow manual adjustment of the mechanical stimuli with the help of diverse external fields such as ultrasonic waves or magnetic fields.^[^ ^87^ ^]^ Thus, real‐time charge‐transfer regulating can be achieved.

Voltage‐gated charge‐transfer channels are also pervasive on the cell membranes. They are sensitive to the membrane potential.^[^ ^74^, ^88^ ^]^ Hence, designing implants that regulate the cell membrane potential are effective in adjusting charge transfer, which can be realized by constructing a charged implant surface. For example, in recent research, bone marrow‐derived macrophages (BMDMs) were cultured on titanium implants with different potential intensities.^[^ ^89^ ^]^ The implant with higher intensity was found to conducive to the upregulation of voltage‐gated potassium ion channel, thus altering the charge transfer and polarization behavior of BMDMs.

It can be found that most the strategies activate the charge‐transfer channels also influence the chemical potential of charges. For instance, the incorporating of metal ions onto the implants leads to a high chemical potential of the introduced ions around the implant, and it also affects the expression of ion channel proteins.^[^ ^83^ ^]^ The methods of regulating cell membrane potential are the same as strategies to adjust the surface potential of implants that we have introduced in Section 3.2.1.^[^ ^10a^ ^]^ Therefore, the charge flow between the cells and the implants is the combined result of the chemical potential and activation of charge‐transfer channels. The relationship between chemical potential and charge concentration or surface potential is well understood, while the roles of chemical, mechanical, or electrical factors in regulating the channel activity are still poorly understood. The activation of charge‐transfer‐related channels is affected by a variety of physiological stimuli, such as ionic gradients, membrane potential, or membrane stress, and their multiple synergy mechanisms are still unclear.^[^ ^42^, ^90^ ^]^ Therefore, at this stage, most implants with manipulating cell transmembrane charge‐transfer abilities are constructed based on the regulation of the electrochemical potential around the implant.

## 4 Advanced Designs of Implants with Charge‐Transfer Monitoring or Regulating Abilities

Many types of devices with charge‐transfer regulating or detecting abilities have been developed and used in neurosurgery, cardiology, orthopedics, stomatology, etc. The neural implant is the most widely applied device for charge‐transfer monitoring or recording in the clinic. Elon Musk's 2020 press conference outlining the progress of his new brain–machine interface (BMI) company, Neuralink, captured public interests worldwide because Neuralink's novel BMI package showed the ability to wirelessly record neurons from pig cortex in real‐time.^[^ ^91^ ^]^ Although this news received numerous attention, the proposed technology is not groundbreaking; a device with similar characteristics has been reported in literature 18 years ago.^[^ ^92^ ^]^ In fact, the BMI developed by Neuralink is just a neural implant with charge‐transfer monitoring and regulating abilities in nature. Generally, the neural implant can be divided into recording devices and stimulating devices.^[^ ^93^ ^]^ Recording devices include microelectrodes such as stereoelectroencephalography electrodes or cortical grids and microelectrode arrays. Simulating devices are mostly in the form of deep brain stimulation (DBS) electrode, which can simulate the target nucleus in the brain, inhibit the electrical impulse of the overexcited neurons; thus, alleviating the symptoms such as tremor, rigidity and, bradykinesia.^[^ ^94^ ^]^ Currently, the DBS has been approved by U.S. Food and Drug Administration (FDA) for the treatment of neuropsychiatric disorders such as Parkinson's disease,^[^ ^95^ ^]^ essential tremor,^[^ ^96^ ^]^ dystonia,^[^ ^97^ ^]^ and obsessive‐compulsive disorder.^[^ ^98^ ^]^ In addition, neural stimulation is promising in auditory^[^ ^99^ ^]^ and visual restoration.^[^ ^100^ ^]^ In cardiology, pacemakers are the most used implant with charge‐transfer‐controlling abilities, it can stimulate the myocardial cell to make the heart excite and contractile; thus, achieving the purpose of treating the heart dysfunction caused by arrhythmias. Smart stents with charge‐transfer‐controlling abilities are also developed.^[^ ^101^ ^]^ It is difficult to know the internal condition and upcoming risks of the traditional cardiovascular stents after implantation. Smart stent which can convey information about its condition, render it possible to check the stent status whenever we want, and treatment to alleviate adverse biological effects can be conducted immediately. To achieve this goal, Chow et al.^[^ ^102^ ^]^ constructed a stent incorporated with a cardiac monitor, which can realize monitoring of cardiovascular disease and identify restenosis of the stent. Takahata et al.^[^ ^103^ ^]^ presented the development of an antenna stent, which can monitor intraluminal flow and pressure. The in situ monitoring abilities of the above‐mentioned smarts stent are realized by incorporating electronic sensors. Researchers have also incorporated electronic sensors into the dental and orthopedic implants to endow the implants with charge‐transfer‐controlling abilities, and realize continuous monitoring of critical intracorporal parameters.^[^ ^104^ ^]^ The obtained data can be used to guide treatments in real‐time and has the potential for massive cost saving to the healthcare system. Such kinds of smart hard tissue implant have been tried to be used for spine fusion,^[^ ^105^ ^]^ fracture fixation,^[^ ^106^ ^]^ knee arthroplasty,^[^ ^107^ ^]^ and dental implant.^[^ ^108^ ^]^ Despite decades of investigation, with very few exceptions, the smart implants for hard tissue have not yet become a part of daily clinical practice, largely because the integration of sensors requires significant modification to the implants, which will deteriorate their mechanical properties.^[^ ^109^ ^]^

It can be found that there are many kinds of implants with charge‐transfer‐controlling abilities that are implanted in different parts of body playing entirely different roles and working in various mechanisms. To cover all types of implantable devices with charge‐transfer‐controlling abilities, we divided the device into three categories according to whether energy input is needed and the types of energy sources used. The first type is the wired implant, which regulates transmembrane charge transfer of cells by changing the surface electric potential through an external circuit.^[^ ^17^ ^]^ The second type is the self‐activated implant, which can create an electrochemical potential gradient in the surrounding environment without external energy input.^[^ ^18^ ^]^ The third type is the stimuli‐responsive implant, which alters the surrounding electrochemical gradient by responding to a wireless field such as light, magnetic, or ultrasonic fields.^[^ ^19^ ^]^ Recent researches on each type of implantable device with transmembrane charge‐transfer‐controlling abilities will be discussed in this section.

### 4.1 Wired Implants

Direct connecting implant with an external circuit is the most common method for transmembrane charge‐transfer regulation and detection.^[^ ^17^ ^]^ Using the implant as an electrode and applying different voltages, the electrochemical potential of the ions and electrons around the implant can be altered, thus inducing an expected transmembrane migration of charges.^[^ ^10a^ ^]^ In addition, the electrochemical potential changes caused by transmembrane charge transfer in cell activities can be detected by the external circuits. Therefore, wired‐implant can help in the real‐time detection of the transmembrane charge transfer.^[^ ^110^ ^]^

The core components of the wired device are the electrode materials in direct contact with biological systems.^[^ ^111^ ^]^ The selection of electrode materials has a crucial influence on the stimulation effect, which determines not only the charge‐transfer mechanism but also the final output current. For specific electrode materials, there is a corresponding limit voltage beyond which hydrolysis reaction will occur, resulting in cell death because of the production of a large number of active substances.^[^ ^112^ ^]^ In addition, an immune response is another factor needed to be considered in the design of wired implants. Upon implantation of foreign matter into the human body, fiber wrapping occurs owing to the mismatch of mechanical and biological properties between the implanted device and the human tissue.^[^ ^113^ ^]^ The insulating fiber layer exhibits high impendence, which will reduce the sensitivity and hurt the performance of the wired devices. Besides, some special requirements on the properties of electrode materials have been proposed for electrodes working in different mechanisms. For the electrode working in the capacitive mechanism, the material should have a high charge storage capacity; for the mechanism of Faradaic charge injection, the material should have a small charge‐transfer resistance.^[^ ^114^ ^]^ Therefore, an ideal electrode should have low resistance and good biocompatibility, cannot cause the death of surrounding cells or produce harmful byproducts during the charge‐transfer process, and be compatible with soft human tissues to prevent fibrosis.^[^ ^115^ ^]^ Recently, various strategies to obtain an ideal wired device have been developed, both in the design of traditional rigid electrodes^[^ ^116^ ^]^ and more advanced flexible electrodes.^[^ ^117^ ^]^

#### 4.1.1 Rigid Electrodes

Wired implants with rigid electrodes are easy to be inserted into human tissue and present high stability. Traditionally used rigid electrodes mainly include Si, Pt, Au, Ir, W, 316 stainless steel, Ti and its alloys, C, and indium tin oxide (ITO).^[^ ^114^ ^]^ Although stainless steel and Ti have good biocompatibility, their surfaces easily form oxidation films, resulting in extremely high charge‐transfer resistance and poor charge injection ability. Inert materials such as Pt, Au, C, and ITO have high electrical conductivity, strong electrochemical stability, good corrosion resistance, and strong charge injection ability in the physiological environment, and they are the most used electrode materials in the clinic. However, rigid electrodes made of bulk materials are hardly used for detecting or regulating the charge transfer of individual cells. Reducing the size of the electrode can improve its spatial resolution, but this causes other engineering problems such as increased impedance and heat dissipation, which reduce the sensitivity of the device and may cause thermal damage to the human body.^[^ ^110^, ^118^ ^]^ Moreover, rigid electrodes made of bulk materials are vulnerable to induce fibrous capsule formation, owing to the mechanical mismatching with human tissue.

In recent years, the development of low‐dimensional materials and nanometer processing technology can address many of these issues.^[^ ^119^ ^]^ Low‐dimensional nanomaterials have a high specific surface area, thus presenting low impedance even at a small size. Their biocompatibility can be easily improved via surface modification. In fact, many human tissues including collagen, bone, and neural networks are built from nanoscale structures.^[^ ^120^ ^]^ Hence, Young's modulus of low‐dimensional nanomaterials is closer to that of the human tissue than that of bulk material. Besides, the nanoscale size provides better spatial resolution, making it possible to monitor and regulate the charge transfer of a single cell. Therefore, various low‐dimensional nanomaterials have been considered as substitutes for the conventional electrodes, including metal‐based nanomaterials such as Au nanoparticles,^[^ ^121^ ^]^ carbon‐based nanomaterials such as C nanotubes^[^ ^122^ ^]^ and graphene,^[^ ^123^ ^]^ and semiconductor‐based materials such as Si nanowires,^[^ ^66^, ^124^ ^]^ gallium phosphide (GaP) nanowires,^[^ ^125^ ^]^ and 2D sulfides.^[^ ^126^ ^]^ Developing new types of low‐dimensional nanomaterials as electrodes has gained significant attention, and various reviews can be found in this field.^[^ ^115^, ^127^ ^]^

**Figure 4:**
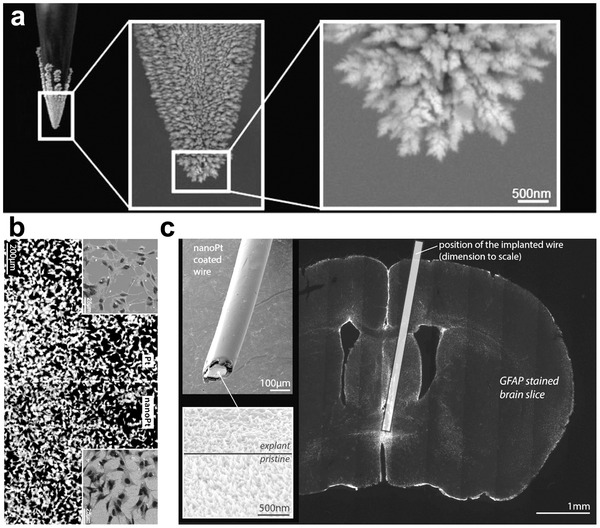
a) Image of electrode with Pt nanostructures on surface. b) Image of Linterna SH‐SY5Y cells cultured on a regular and nano‐Pt surface. The inset figures show SEM images of the cells on both surfaces, demonstrating homogenous distribution of interconnected cells with similar morphology, suggesting equivalent biocompatibility of both substrates. c) Glial fibrillary acidic protein (GFAP)‐stained brain tissue slice visualizing the extent of the inflammatory processes caused by the implanted stimulation wire inside the brain. The position of the wire is indicated (to scale) by the rectangle in the image. The wire was explanted prior to histological tissue preparation and imaged by SEM to evaluate the coating status. The insets show the entire wire and a representative image of the coating of an explanted electrode in comparison to a pristine coating. Despite some tissue remaining on the explanted wire, it is clear that the surface was not damaged, illustrating the high mechanical and electrochemical stability of the nano‐Pt coating. Reproduced with permission.^[^ ^128^ ^]^ Copyright 2020, American Chemical Society.

Additionally, the nanostructures can be directly constructed on the surface of the conventional rigid electrode to ensure the strength of the electrode for insertion, and achieve good mechanical matching of the electrode to the contact part of the human tissue. Surface nanoritization is an effective way of reducing surface hardness. Moreover, as the specific surface area of the electrode increases, the charge injection capability improves. For example, nanostructured platinum was constructed on Pt electrode via electrochemical deposition, as seen in **Figure** **4**a.^[^ ^128^ ^]^ Compared with the conventional Pt electrode, electrodes with Pt nanostructures present reduced impedance and good biocompatibility, thus promoting cell growth and adhesion (Figure 4b). In addition, the nanoscale structured surface has the ability to reduce the incidence of electrode encapsulation (Figure 4c); thus, presenting outstanding long‐term stability for recording and stimulation. Nanostructures have also been fabricated on other types of rigid electrodes including Ir and Au, and both Ir and Au electrodes with nanostructures have lower impedance and higher biocompatibility than their untreated counterparts.^[^ ^129^ ^]^ However, some studies have pointed out that low‐dimensional nanomaterials may induce carcinogenic and teratogenic effects,^[^ ^130^ ^]^ and thus the biosafety of these materials has yet to be tested before clinical trials.^[^ ^131^ ^]^

#### 4.1.2 Flexible Electrodes

**Figure 5:**
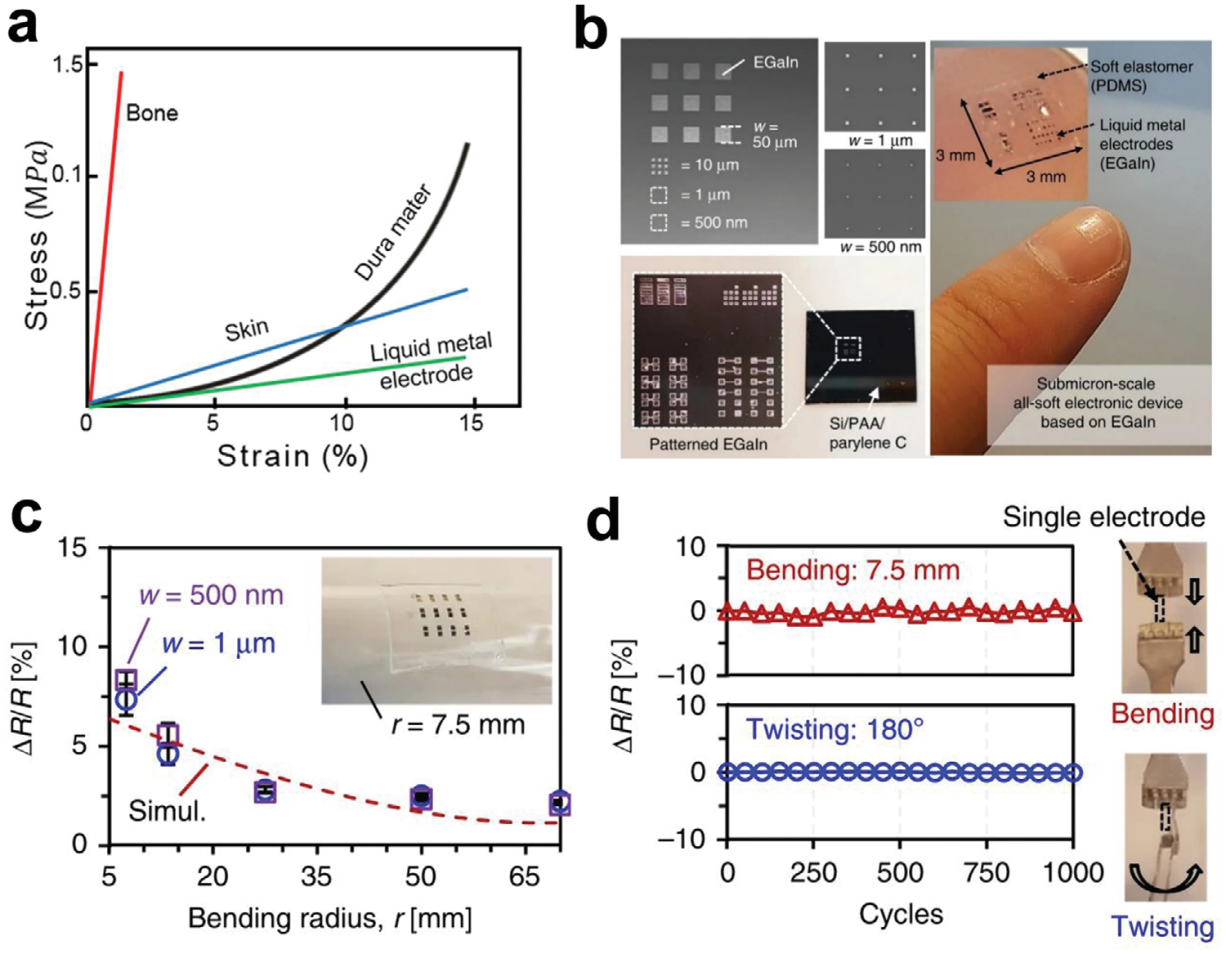
a) Strain–stress curves of liquid metal electrodes, other metal electrodes, and biological tissue. b) Patterned EGaIn square‐shaped dot arrays with various dot dimensions from 50 µm down to 500 nm, soft material encapsulation, and release process of the EGaIn structures. c) Measured and simulated relative resistance changes Δ*R*/*R* of the resistive strain sensors as a function of the bending radius. d) Measured relative resistance change as a function of the number of bending and twisting cycles (bending was performed around a cylinder with 7.5 mm radius, and twisting was performed to an 180° angle). a) Reproduced with permission.^[^ ^135^ ^]^ Copyright 2017, IOP Publishing Ltd. b–d) Reproduced with permission.^[^ ^136^ ^]^ Copyright 2020, Nature Publishing Group.

Conformability is an essential prerequisite for steady and reliable charge‐transfer monitoring and regulating. It depends not only on Young's modulus of the device material but also the relation between the tissue curvature at the implantation site and the corresponding device's thickness and geometry, which define the moment of inertia and the interactions at the material‐tissue interface.^[^ ^132^ ^]^ The conventional rigid planar wired device is difficult to fit in the human tissue completely, specifically owing to the complex soft environment of organisms. Flexible electronic devices developed in recent years are expected to solve this problem.^[^ ^133^ ^]^ Generally, the flexible electronic devices use biocompatible polymers such as polyimide (PI) and polydimethylsiloxane (PDMS) as substrates, and a noble metal as an electrode.^[^ ^134^ ^]^ However, noble metals have high elastic modulus, low charge storage capacity, and low charge injection limit. To meet the mechanical requirements of the biological tissue and realize functionalization simultaneously, liquid metal has attracted the attention of researchers. Liquid metals are highly ductile and conductive, and the most common liquid metals used in biological applications are Ga–In alloys. At room temperature, the Ga–In alloy exhibits zero stiffness and nearly infinite ductility owing to its fluidity (**Figure** **5**a), which can adapt well to the soft mechanical environment of biological tissue.^[^ ^135^ ^]^ Kim et al.^[^ ^136^ ^]^ developed an all‐soft electrode array based on eutectic Ga–In alloy (EGaIn) (Figure 5b). Owing to the intrinsically soft properties of the EGaIn, the fabricated device can maintain its electrical functionality even after 1000 bending or twisting cycles, and it endures mechanical strain >30% as well as folding deformation (Figure 5c,d). Moreover, the patterned electrode presented a feature size as small as 180 nm, and the high resolution provides high potential that can be applied in the detection and stimulation of the single cell.

Conductive polymer (CP) is another ideal material for soft wired devices. CP has high conductivity, high specific surface area, and good stability.^[^ ^137^ ^]^ The electrical performance of the CP can vary greatly with the degree of doping, from insulator to conductor. At present, common CPs include polypyrrole (PPy), poly(3,4‐ethylenedioxythiophene):polystyrene sulfonate (PEDOT:PSS), polyaniline, and polythiophene, and their biocompatibility can be enhanced further by doping different materials.^[^ ^138^ ^]^ In a recent study, PPy containing the anionic dopant dodecylbenzenesulfonate was selected as an electrode to stimulate the human induced pluripotent stem cells (iPSCs), and the results showed that the PPy electrode presented high biocompatibility and the PPy‐mediated electrical stimulation could promote differentiation of the iPSCs.^[^ ^139^ ^]^ In another work, Rizau‐Reid et al.^[^ ^140^ ^]^ improved the functionality of PEDOT:PSS in neural tissue engineering by incorporating 3,4‐ethylenedioxythiophene (EDOT) oligomers, and then constructed an electroactive and biocompatible block copolymer. The neurite length and branching of neural stem cells can be enhanced on the prepared material under electrical stimulation, indicating the potential of these materials to be used to construct soft electrodes. In addition to CP, conventional polymers can become conductive by mixing with conductive materials. Deng et al.^[^ ^141^ ^]^ introduced graphene oxide (GO) into a poly(vinyl alcohol) (PVA) hydrogel and the GO–PVA hydrogel was modified further to obtain bioadhesive capability. The prepared hydrogel composites could be coated on bioelectric implants, constructing a conformal, stable, and conductive interface with wet and soft biological tissue.

**Figure 6:**
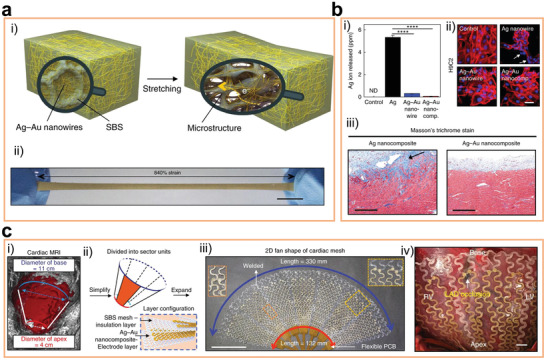
a‐i) Schematic illustration of the microstructured Ag–Au nanocomposite before and after stretching, and a‐ii) optical camera image of the microstructured Ag–Au nanocomposite after being stretched. ICP‐MS analysis of Ag ions released from Ag nanowires, Ag–Au nanowires, and the Ag–Au nanocomposite after incubating each in culture medium for 3 days. b‐i) Low levels of Ag ions for Ag–Au nanowires and Ag–Au nanocomposite show that the Au sheath effectively protects Ag nanowires from dissolution. ND, not detected. b‐ii) Confocal microscope image of H9C2 cells after exposure to original medium (control) or medium extracts of Ag nanowires, Ag–Au nanowires, and Ag–Au nanocomposite for 24 h. Cells exposed to Ag nanowire extracts exhibit damaged (arrows) actin cytoskeleton (red) and DNA (blue). Scale bar, 50 µm. b‐iii) Masson's trichrome staining of cardiac muscles after 3 weeks implantation of the Ag–Au nanocomposite shows less fibrotic reaction and inflammatory response than those implanted with Ag nanocomposite. c‐i) 3D cardiac magnetic resonance image of a swine heart (red). c‐ii) Schematic illustrating the design process for the cardiac mesh. The shape of the heart is simplified as a cone frustum, which is unfolded into a 2D fan shape consisting of seven repetitive segments welded together. Inset: Cardiac mesh consists of two electrode layers (Ag–Au nanocomposite) and three insulation layers (SBS). c‐iii) Optical camera image of a cardiac mesh and c‐iv) implanted cardiac mesh on a swine heart. Reproduced with permission.^[^ ^127e^ ^]^ Copyright 2018, Nature Publishing Group.

Structural design has a decisive effect on the performance of the charge‐transfer monitoring or regulating device. Except for the intrinsic tensile properties of the flexible material itself, the rigid material can be stretched through designing an appropriate geometric structure. Kirigami is an effective method for constructing flexible devices. This is the construction of 2D or 3D structures from a piece of paper or film through folding, cutting, and gluing.^[^ ^142^ ^]^ In a previous report, an electrode with a highly flexible structure was fabricated via kirigami. Although the electrode was composed of unstrechable Pt/Ti and parylene, the fabricated device presented a strain as high as 840% under a stress of 0.53 MPa.^[^ ^143^ ^]^ Similarly, controlling the assembly of 1D wires through weaving can obtain an advanced stretchable electrode.^[^ ^144^ ^]^ Because of their high conductivity, Ag nanowires (AgNWs) are the commonly used 1D materials to fabricate electrodes. Microelectrodes assembled by AgNW showed high flexibility, and no bucking and fracture were observed after rolling at a bending radius of ≈5 mm for 100 cycles.^[^ ^145^ ^]^ However, Ag ions released from the AgNWs may cause biosafety concerns. Choi et al.^[^ ^127e^ ^]^ designed an Ag–Au nanocomposite that by combining a mixture of Ag–Au core–sheath nanowires, styreneic block copolymers (SBSs) elastomer, and an additional hexylamine in toluene (**Figure** **6**a), and it could withstand a strain as high as 840%. Owing to the Au encapsulation, the release of Ag ions was inhibited, and the composite presented high biocompatibility (Figure 6b). A large‐area soft cardiac mesh for recording and stimulating at multiple locations of the swine heart was constructed based on the Ag–Au nanowire composites (Figure 6c). In vivo experiments showed that 30% of cyclic stretching cannot change the performance of the cardiac mesh, verifying its high flexibility.

Wired implant devices can monitor and regulate the transmembrane charge‐transfer process in a dynamic manner with high precision. However, the requirement of an external power supply brings obvious limitations, including the potential for infection, mobility of the patient, and patient acceptability.^[^ ^146^ ^]^ Replacing external power supply with implanted batteries is able to go beyond the above limitations. Battery‐powered systems provide direct power to the implanted implants and do not have the disadvantages of percutaneous lead. However, battery‐powered implants are limited by their large size, owning to the accommodation of bulk battery, and the finite life of battery. Therefore, developing wireless chargeable battery in small size and with high biocompatibility is expected to improve the effectiveness of clinical application of wired implantable devices.^[^ ^147^ ^]^ Moreover, the wired implant devices are vulnerable to fiber wrapping because of their mismatched mechanical and biological properties, which will decrease their sensitivity and shorten their working life. Therefore, developing nanosized or flexible electrodes with good biocompatibility, high charge injection ability, and good mechanical property matching with the human tissues are crucial to enhance the performance of wired implant devices.

### 4.2 Self‐Activated Implants

**Figure 7:**
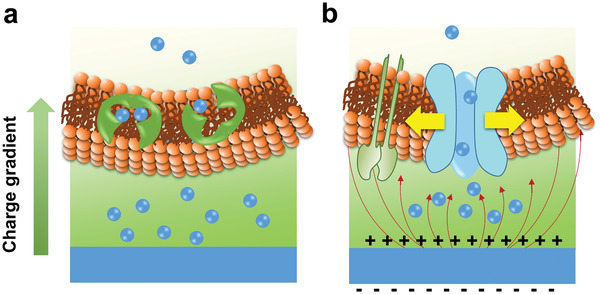
Strategies to control charge transfer between implant and organism in a self‐activated manner: a) controlling charge gradient around the implant, and b) controlling the surface potential of the implant.

During the regulation process, it is necessary for wired biomedical implant devices to connect with a power source that increases the system complexity and brings safety concerns. Therefore, several strategies have been developed to manipulate the charge transfer in a self‐activated manner, including controlling the charge gradient around the implant (**Figure** **7**a), and constructing an implant surface with controlling surface potential (Figure 7b).

#### 4.2.1 Implants Controlling Local Charge Gradient

**Figure 8:**
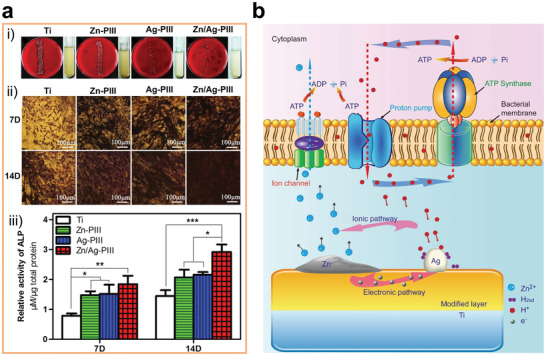
a‐i) Roll‐over cultures obtained from explanted implants after incubation for 24 h. The insets are implants immersed in culture medium. a‐ii) ALP positive areas of rBMSCs cultured on various surfaces for 7 and 14 days and a‐iii) corresponding colorimetrically qualitative results. b) Schematic illustration of the possible antibacterial mechanism on the Zn/Ag coimplanted titanium surface. Reproduced with permission.^[^ ^155^ ^]^ Copyright 2014, Elsevier.

Constructing a film or coating containing active elements is an effective strategy to endow implant materials with biological functions. The release of active ions from the implanted materials creates a local microenvironment with high ion concentration. The high extracellular ion concentration can increase the transmembrane ion gradient and generate a driving force for ions to enter the cell. Thus, the uptake of active ions by the cells can be increased to realize positive regulation of cell behavior. The physiological regulation by ion transfer between the implant materials and the cells depends on two aspects. The first one is the selection of the doping active ions because different types of ions have multifarious physiological functions. For instance, Ca,^[^ ^148^ ^]^ Sr,^[^ ^149^ ^]^ and Mg^[^ ^150^ ^]^ ions have the ability to promote bone formation, Cu ions present preferable angiogenesis effects,^[^ ^151^ ^]^ and Ag ions are widely recognized as antibacterial agents.^[^ ^152^ ^]^ To meet the different clinical demands, many implants containing different active elements were designed through various techniques, and the regulation of cell behavior through the transfer of active ions between the implant materials and the cells was successfully achieved. In addition to single‐ion doping, codoping ions with different physiological functions can endow the implant materials with multiple physiological functions. There may be a synergistic effect between different ions,^[^ ^153^ ^]^ and multi‐ion doping may show better comprehensive physiological effects than single‐ion doping.^[^ ^154^ ^]^ For instance, a Ti surface codoped with Ag and Zn possesses both the antibacterial ability of Ag and the osteogenic effect of Zn (**Figure** **8**a).^[^ ^155^ ^]^ Additionally, the galvanic corrosion pair formed by the codoped Ag and Zn can accelerate the release of Zn ions (Figure 8b), achieving a better osteogenic effect than the single Zn‐doped Ti.^[^ ^156^ ^]^ Similar synergistic effect was also observed for Ca/Sr,^[^ ^157^ ^]^ Zn/Mg,^[^ ^158^ ^]^ Cu/C,^[^ ^159^ ^]^ Cu/N,^[^ ^160^ ^]^ Ag/Ca,^[^ ^161^ ^]^ and Ag/Mg^[^ ^162^ ^]^ codoped Ti implants. A controlled ion release rate is the other key factor to endow the implant materials with specific physiological functions. The physiological functions of ions are dose‐dependent, and most active ions exhibit certain toxicity at high concentrations.^[^ ^163^ ^]^ Although the released ions may have high biocompatibility in the usual sense, the surrounding cell will die from dehydration if the ion release rate is too fast. For instance, the rapid degradation rate of Mg alloys causes a high concentration of the local Mg ion, which easily results in the death of nearby cells. Hence, limiting the ion release rate of Mg‐based biomaterials to a safe range is a major direction promoting their clinical application.^[^ ^164^ ^]^ The easiest way to change the element release rate is to adjust the doping amount. However, the functional concentration of ions is on the micromolar scale.^[^ ^165^ ^]^ It is still a great challenge to achieve precise regulation of the ion release amount at such a low level. On the other hand, the ion release rate is related to the existing form of the element, which dominates their stability in physiological environments, and the construction of compounds with lower solubility decreases the ion release rate significantly. The sulfide stability of elements such as Ag and Zn is much higher than their oxides; therefore, sulfuration of implanted materials containing the elements can effectively reduce the negative effects caused by excessive ion release on the premise of preserving their physiological functions.^[^ ^166^ ^]^ Changing the contact area between the active elements and the environment is another way of controlling their release rates. After doping with functional ions, a biocompatible barrier layer can be further constructed on the material surface, which can effectively limit the ion release rate and prevent the sudden release of ions, thus exerting a long‐term physiological effect.^[^ ^167^ ^]^

**Figure 9:**
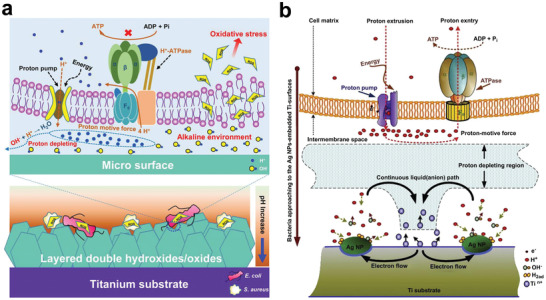
a) Illustration for the possible antibacterial mechanism of the local alkaline microenvironments generated by LDH/LDO films on titanium surface. b) Illustration for the possible toxicity mechanism on the Ag nanoparticles embedded surfaces. a) Reproduced with permission.^[^ ^168a^ ^]^ Copyright 2018, American Chemical Society. b) Reproduced with permission.^[^ ^170a^ ^]^ Copyright 2011, Elsevier.

Another strategy of changing ion exchange between the implant material and the biological system is to construct a surface that can directly react with specific ions in the physiological environment. Through selectively consuming various types of ions, the original ion concentration gradient can be reduced, thus changing the direction of ion transfer. Constructing an alkaline film on the implant materials is an example of this strategy. The alkaline surface can selectively consume H ions in the environment, resulting in a local H ion depleted area.^[^ ^168^ ^]^ It is essential for bacterial ATP synthesis to maintain a high H ion transmembrane gradient. Conversely, the ATP synthase of the eukaryotic cells is located in the mitochondria of the cell, and the eukaryotic cells are less affected by changes in extracellular H ion concentration. Therefore, the implant materials with proper alkalinity present a selective antibacterial effect (**Figure** **9**a).^[^ ^169^ ^]^ During electrochemical corrosion, the following reactions occur in the metal‐based implant materials

(3) $$2H^{+}+2e^{-}=H_{2}↑$$

(4) $$O_{2}+2H_{2}O+4e^{-}=4OH^{-}$$

The above reactions can also effectively consume H ions, thus achieving a similar selective antibacterial effect as that of alkaline surfaces. When a second conductive matter was introduced on the surface of the metal implants, the cathodic reaction and anodic reaction separated, which accelerated the electrochemical reaction and H ion consumption, thus endowing the implant with better antibacterial effects (Figure 9b).^[^ ^170^ ^]^

#### 4.2.2 Implants with Controlled Surface Potential

Introducing permanent charges on the implant surface will produce an internal electric field around the implant and thus affecting the charge transfer. The following are several methods for obtaining an implant with charged surfaces.

Coating ferroelectric material on the surface of the implant or directly using ferroelectric material as the implant is the most effective method of introducing permanent charges on the surface of the implant.^[^ ^171^ ^]^ Ferroelectricity is the spontaneous polarization of a material within a certain temperature range. The positive and negative charge centers in the ferroelectric lattice do not coincide, resulting in the formation of an electric dipole moment even without applying an electric field, and the spontaneous polarization direction changes along the external electric field.^[^ ^172^ ^]^ Owing to its excellent ferroelectric properties, lead zirconate titanate (PZT) is the most used piezoelectric material in the biomedical field. Studies have shown that the axons of rat cortical neurons cultured on the surface of PZT are significantly prolonged, and the nerve activity is also obviously improved.^[^ ^173^ ^]^ However, PZT contains high amounts of lead (60 wt%), which may cause serious health problems and pose safety risks.^[^ ^174^ ^]^ Therefore, significant attention has been paid to Pb‐free piezoelectric materials in recent years. Both inorganic (such as zinc oxide (ZnO),^[^ ^175^ ^]^ barium titanate (BaTiO_3_, BTO),^[^ ^176^ ^]^ potassium sodium niobate (KNN),^[^ ^177^ ^]^ lithium sodium potassium niobate (LNKN),^[^ ^178^ ^]^ boron nitride nanotubes (BNNTs),^[^ ^179^ ^]^), and organic (such as polyvinylidene difluoride (PVDF) and its copolymers and biopolymers^[^ ^180^ ^]^) materials have been widely employed in bone,^[^ ^181^ ^]^ muscle,^[^ ^182^ ^]^ and nerve stimulation or reparation.^[^ ^18^, ^183^ ^]^ There are various good reviews in this area, and we will not repeat them here. We will pay more attention to how ferroelectric materials affect the charge transfer between materials and cells.

Charges exist on the surface of the polarized ferroelectric materials, which result in a potential difference (namely, electric field) between the surface of the implant and the physiological environment. The formed electric field affects the potential of the cell membrane, leading to a change in the conformation of the voltage‐gated ion channels existing on the cell membrane. This affects the ion exchange between cells and materials. Currently, the specific effects of the built‐in electric field on ion channels are still controversial. It is generally believed that the transfer of Ca^2+^ ions are affected by the action of electric fields. Studies have shown that the electric field causes the rearrangement of intracellular charges, which leads to the opening of Ca^2+^ion channels. The phenomenon increases the uptake of Ca^2+^ ions by cells, which can regulate a series of cell behaviors further. This is consistent with the finding that the concentration of Ca^2+^ ions in the neurons cultured on the PVDF surface could be significantly increased and the axons of the neurons were significantly prolonged.^[^ ^184^ ^]^

Except affecting the “on” and “off” state of the ion channel, the built‐in electric field causes directional movement of ions in the environment, resulting in the changes of ion concentration around the cell, which ultimately changes the uptake of the ions by cells. Notably, the ferroelectric surface with negative charges could induce the accumulation of Mg^2+^ and Ca^2+^ in the environment into cells, causing the implant material to exhibit a better osteogenic effect.^[^ ^185^ ^]^ When the ferroelectric material is doped with functional elements, the formed electric field can also promote the enrichment of the doped ions to the cells, thus endowing the implanted material with more physiological functions. Zhai et al.^[^ ^186^ ^]^ prepared a copper oxide‐doped KNN, and the Cu ions accumulated in the bacteria under the action of the electric field formed by KNN, presenting good antibacterial capacities. Similarly, Se, which is an effective anticancer element, was doped into the KNN piezoceramic, realizing the wireless combination of electrotherapy and chemotherapy.^[^ ^187^ ^]^

**Figure 10:**
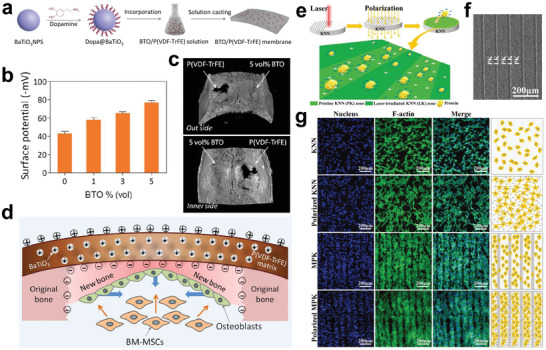
a) Schematic diagram of BTO NP/P(VDF‐TrFE) membrane fabrication process. b) The surface potential of polarized nanocomposite membranes containing different amounts of BTO. c) Representative micro‐CT images and sagittal view images of critical‐sized rat calvarial full‐thickness defects at 12 weeks postimplantation. Blue arrows denote the residual membrane materials. Yellow arrows denote the regenerated new bone. Yellow dotted lines denote the boundary between nascent bone and host bone. Results show good bone defect repair abilities in rat calvarial models after implantation of polarized nanomembranes with 5 vol% BTO content and polarized neat P(VDF‐TrFE) membranes. d) Illustration of biomimetic electric microenvironment created by BTO NP/P(VDF‐TrFE) composite membranes encouraging bone defect repair. Electrical dipoles of BTO NPs are reoriented in the direction of poling electric field after corona poling treatment, and consequently induced charges are generated on the outer surface of the membrane. When the composite membranes are implanted like native periosteum covering the bone defect, endogenous bone marrow mesenchymal stem cells (BM‐MSCs) can be recruited by galvanotaxis and induced to differentiate into osteoblasts. Consequently, the membranes sustainably maintained electric microenvironment giving rise to rapid bone regeneration and complete mature bone‐structure formation integrated with original bone. The short black arrows denote the direction of electrical dipole in BTO NPs. The blue thick arrows denote the direction of new bone growth. The orange thin arrows denote the recruitment and osteogenic differentiation of BM‐MSCs. e) Schematic illustration of site‐selective protein adsorption regulated via a space charge model. The space charge model, the microdomain charge distributed piezoelectric K_0.5_Na_0.5_NbO_3_ (MPK), was prepared by a laser‐induced phase distribution change. Laser irradiation decreased the piezoelectricity of the microdomains. Thus, after polarization, the two zones with different piezoelectricities showed significant differences in charge density, and more positive charges were generated in the pristine KNN (PK) zone than in the laser‐irradiated KNN (LK) zone, leading to a nonuniform spatial distribution of the charge density. f) SEM images of the MPK surface (scale bar = 200 µm); the PK zone and LK zone showed a periodic spatial distribution. g) Characterization of cellular adhesion, spreading, and orientation. Fluorescence images of a high density of cells on different samples after culturing for 24 h; F‐actin was stained with FITC (green), and nuclei were stained with DAPI (blue) (scale bar = 200 µm). The schematic describes the behavioral characteristics of cell growth on the surface of the corresponding sample. a–d) Reproduced with permission.^[^ ^188^ ^]^ Copyright 2016, American Chemical Society. e–g) Reproduced with permission.^[^ ^189^ ^]^ Copyright 2019, The Royal Society of Chemistry.

The intensity and distribution of the constructed electric field are the key factors affecting the ion transfer between the material and the cells. The electric field intensity can be adjusted by regulating the polarization electric field intensity or changing the composition of the ferroelectric materials. Zhang et al.^[^ ^188^ ^]^ prepared a charged implant by mixing BTO nanoparticles into a poly(vinylidene fluoridetrifluoroethylene) (P(VDF‐TrFE)) matrix (**Figure** **10**a). The surface potential of the nanocomposite membranes could be regulated by optimizing the composition ratio. When the BTO nanoparticle content was 5%, the membrane exhibited a surface potential of −76.8 mV (Figure 10b). The membranes can sustainably maintain the electric microenvironment in vivo, giving rise to rapid bone regeneration (Figure 10c,d). The distribution of the electric field is generally regulated via patterning, and it has an important effect on cellular behavior. Using laser processing technology, ferroelectric materials with alternating polarization could be constructed, thus creating a periodic electric field (Figure 10e,f). The patterned internal electric field could enhance cell adhesion and filamentous formation, promoting cell‐oriented arrangement and accelerating cell migration further (Figure 10g).^[^ ^189^ ^]^

Creating interface potential differences is another strategy to regulate the surface potential. The aquatic environment in the organism is a conductive phase, and the commonly used implant materials are mostly metals with the ability to conduct electricity.^[^ ^190^ ^]^ The transition of potential from one conductive phase to another occurs entirely at the phase interface.^[^ ^191^ ^]^ A significant potential change will generate a strong electric field at the interface, which can be expected to have a great influence on the behavior of the charge carriers (electrons or ions) in the interface region, thus controlling the direction and rate of charge transfer.^[^ ^192^ ^]^ The potential of the interface can be regulated by designing a specific implant surface and interface. Charge transfer between the material and the organism changes along with the potential of the interface, and then it regulates the cellular behaviors.^[^ ^193^ ^]^ The interface potential difference is mainly caused by the difference between the Fermi energy level of the material and the endogenous redox energy level of the physiological environment. Studies have shown that the endogenous biological redox potential (BRP) is ≈−4.12 to −4.84 V in vivo,^[^ ^194^ ^]^ and materials with energy levels outside this range can easily exchange charges with the physiological environment.^[^ ^195^ ^]^ Based on this, Burello and Worth^[^ ^195^, ^196^ ^]^ proposed a theoretical model to predict the toxicity of material oxides by comparing material energy levels and BRP. Although more experiments need to be conducted to verify the theory, it points out a possible way for designing biomaterials.^[^ ^197^ ^]^ Currently, two strategies are widely used to regulate the potential of the interface.

**Figure 11:**
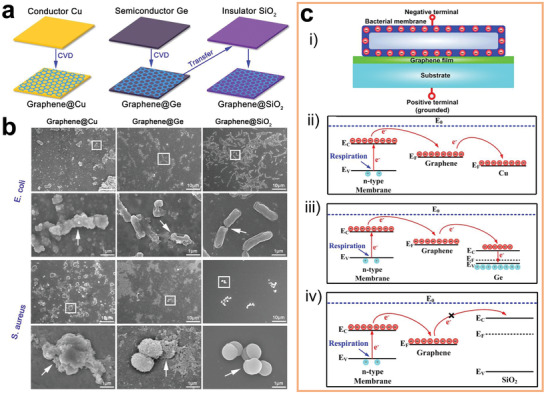
a) Schematic illustration for the fabrication of the graphene film samples, i.e., large‐area monolayer graphene films on conductor Cu, semiconductor Ge, and insulator SiO_2_ substrates. b) SEM morphology of the *E. coli* (top panel) and the *S. aureus* (bottom panel) that were seeded onto the graphene films at both low and high magnifications, with the seeded concentration of bacteria being 10^7^ CFU mL^−1^. The white arrows at high magnification correspond to the rectangular areas at low magnification, respectively. Schematic circuitry to illustrate the proposed mechanism for the observed phenomena of different responses of bacteria to the graphene films in c‐i) darkness on c‐ii) conductor Cu, c‐iii) semiconductor Ge, and c‐iv) insulator SiO_2_ substrates from the view of the energy band diagrams of these graphene‐on‐substrate junctions. Reproduced with permission.^[^ ^198^ ^]^ Copyright 2014, Nature Publishing Group.

The first method is to change the electrical contact mode, including insulating contact, Schottky contact, and ohmic contact. In the insulating contact, charges cannot run across the interface. In the Schottky contact, the charge transfer needs to pass through the Schottky barrier. In the ohmic contact, the interface resistance is small, and thus the charges can run across the interface easily. In a previous study, we deposited a graphene film on the surfaces of conductors (Cu), semiconductors (Ge), and insulators (silicon dioxide), constructing three surfaces with different electrical contact modes (**Figure** **11**a).^[^ ^198^ ^]^ The results indicated that the graphene film on the surface of Cu had the strongest antibacterial effect, followed by that on the surface of Ge, and the graphene on the surface of insulating silicon dioxide presented no significant antibacterial effect (Figure 11b). We attributed the antibacterial ability of graphene on the Cu surface to its strong electrical conductivity stemming from the ohmic contact, which enables the electrons in the bacterial membrane to be transferred to the material, leading to the destruction of the bacterial respiratory chain (Figure 11c). For silica, its insulating contact prevents electron transfer between the bacteria and the material; therefore, it has no antibacterial properties. The conclusion is still controversial; some studies proved the above conclusion with a wider range of substrate selection, and they verified that the contact modes affect their antibacterial ability.^[^ ^199^ ^]^ However, different views toward this issue also exist. Dellieu et al.^[^ ^200^ ^]^ argued that the antibacterial abilities of the graphene‐coated Cu samples resulted from the release of Cu ions. They designed a graphene‐coated Au platform, which also formed an ohmic contact, but it did not show any antibacterial effect. This experimental result contradicted the theory that contact mode would endow the implants with antibacterial abilities. It can be seen that the influence of the electrical contact mode on the charge transfer between the material and the physiological environment and the cell behavior is unclear so far.^[^ ^201^ ^]^ More advanced testing methods, that can be used to observe electron transfer between the material and the bacteria intuitively, are required to clarify the mechanism.

**Figure 12:**
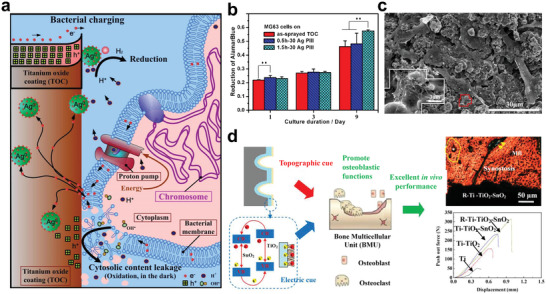
a) Illustration for extracellular electron transfer stimulated biocide action of Ag/titanium oxide coating (TOC) composites in the dark. That is, electrons are transferred from the bacterial membranes to the TOC surface, stored on the Ag NPs (“bacterial charging”), and induce valence‐band hole (h^+^) accumulation at the TOC side that explains cytosolic content leakage. b) Reduction of AlamarBlue for MG63 cells cultured for periods of time on various surfaces, cell density in the suspension is ≈1 × 10^5^ cell mL^−1^, and c) SEM morphology of the MG63 cells cultured for 1 day on Ag/TOC composite, with the higher magnification image of the circled area in the inset of panel (c). d) Illustration of the enhanced osteogenesis performance of titanium by an electric cue offered by the built‐in electrical field of SnO_2_–TiO_2_ heterojunction and the topographic cue provided by the hierarchical surface structure. a–c) Reproduced with permission.^[^ ^209a^ ^]^ Copyright 2013, Elsevier. d) Reproduced with permission.^[^ ^212^ ^]^ Copyright 2018, American Chemical Society.

The second way to regulate the interfacial potential is to change the band structure. TiO_2_ has good biocompatibility and is an n‐type semiconductor. At present, the band engineering of implant materials is mostly based on TiO_2_ thin films, which are mainly obtained by doping or constructing heterogeneous nodes. TiO_2_ films doped with N,^[^ ^202^ ^]^ C,^[^ ^203^ ^]^ H,^[^ ^204^ ^]^ O,^[^ ^205^ ^]^ and other elements^[^ ^206^ ^]^ can be constructed via chemical or physical synthesis. The energy gap of TiO_2_ changes after doping it with the elements, making its energy level move with respect to BRP and thus regulating the charge transfer between the material and the living system. The construction of a heterogeneous junction can also regulate the energy band structure of implant materials. A variety of methods have been developed to load metal nanoparticles such as Au,^[^ ^207^ ^]^ Pt,^[^ ^208^ ^]^ Ag,^[^ ^209^ ^]^ Fe,^[^ ^210^ ^]^ and Co^[^ ^193^ ^]^ to the surface of TiO_2_ film. Because the Fermi level of TiO_2_ and the metal particles are different, the energy band of TiO_2_ will bend and form a potential barrier after contact with the metal particles, namely, the Schottky barrier. This barrier can promote the accumulation of electrons in the bacterial membrane into the materials and destroy the electron transport chain on the membrane. In addition, it can inhibit the recombination of holes and electrons in the materials. The incomposite holes in the materials can further migrate to the surface of the material and react with water to form free radicals, which enhances the antibacterial effect further. Therefore, the TiO_2_ films embedded with the metal nanoparticles exhibited better antibacterial capacities (**Figure** **12**a).^[^ ^211^ ^]^ Since the respiratory chain of the eukaryotic cells is located on the mitochondria membrane inside the cell, the cell cannot exchange electrons with the material directly; hence, the designed platforms presented little adverse effect on the cells (Figure 12b,c).

**Figure 13:**
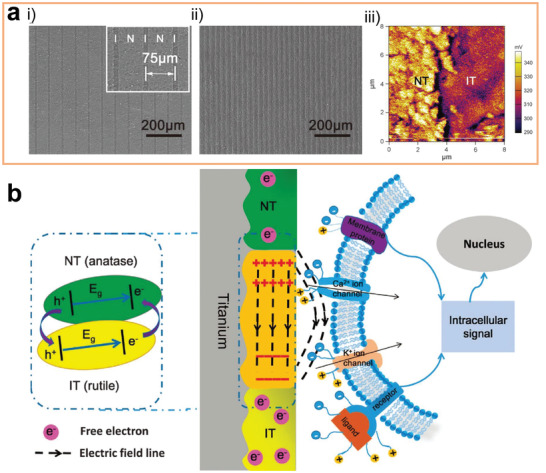
Construction of the microscale electrostatic field (MEF). SEM images of constructed MEFs with different charged domain intervals: a‐i) 75 µm interval and a‐ii) 30 µm interval on the titanium surface. N refers to NT (nonirradiated n‐type semiconducting anatase TiO_2_ zone prepared by hydrothermal synthesis), and I refers to IT (n‐type semiconducting rutile TiO_2_ zone prepared by laser irradiation of the anatase TiO_2_ zone). The inset is the magnified view of the SEM image. a‐iii) Kelvin probe force microscopy (KPFM) image of the border of the NT (left) and IT zones (right) of the MEF sample with a 30 µm interval showing that the relative potential on the NT zone is ≈19 mV greater than that on the IT zone. b) Illustration of the mechanism used to generate the microscale electrostatic field (MEF) and the interaction between the MEF and the stem cells. The diagram in the dashed line box illustrates the TiO_2_ phase junction of the NT and IT, as well as the electron transfer from the NT (rutile) to the IT (anatase) zone. (Right) Stem cell membrane with charged protein affected by MEF. As ion channels, membrane proteins, ligands, and receptors are all charged with different surface potentials, their surface charges would be polarized under the guidance of MEF. The sustained built‐in MEF enables the polarized stem cell surface species to transduce signals to the nucleus to activate osteogenic genes that results in enhanced osteogenesis. Reproduced with permission.^[^ ^214^ ^]^ Copyright 2016, Nature Publishing Group.

An internal electric field also can be formed by constructing a heterojunction with the Schottky barrier. As described in the previous section, the built‐in electric field induces ion exchange between the material and the cell, mediating different biological effects. Zhang and co‐workers^[^ ^212^ ^]^ fabricated a SnO_2_–TiO_2_ heterojunction on the surface of Ti. The electric signal provided by the Schottky barrier and the topographic cue provided by the hierarchical surface structure could significantly improve the osteogenic function of the cells around the implant (Figure 12d). In another work, we constructed a layered double hydroxides (LDHs)–TiO_2_ heterojunction, which promoted the transfer of holes in materials to the physiological environment, enhancing the antibacterial effect of the implant.^[^ ^213^ ^]^ Similar to piezoelectric materials, periodic electric fields can also be constructed by using heterogeneous junctions. The energy levels of TiO_2_ with different crystalline phases are different. From this perspective, Ning et al.^[^ ^214^ ^]^ constructed a periodically distributed anatase/rutile junction on a Ti surface via laser processing (**Figure** **13**a). The constructed platform showed a patterned microscale electric field, resulting in an effective electrical cue that promotes the transfer of charge between the material and the cells. In vitro and in vivo studies have demonstrated that the microscale electric field induces osteogenic differentiation of stem cells and promotes bone regeneration around the implant (Figure 13b).

**Figure 14:**
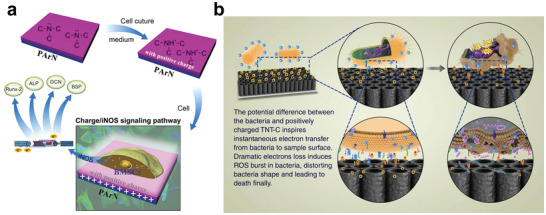
a) Mechanical illustration of the positively charged surface with tertiary amines upregulating osteogenic differentiation of BMSCs via the iNOS pathway signaled by the surface charge. b) Diagram showing antibacterial mechanism of charged titania nanotubes doped with carbon (TNT‐C). a) Reproduced with permission.^[^ ^215^ ^]^ Copyright 2015, Nature Publishing Group. b) Reproduced with permission.^[^ ^217^ ^]^ Copyright 2018, Nature Publishing Group.

There are some other strategies for introducing permanent charges on the surface of the implant to regulate the surface potential. For instance, an electric field can be built by modifying the implant materials with charged polymers. The intensity of the electric field can be controlled by adjusting the grafting amount of the charged polymer. A positively charged surface with controllable tertiary amine was obtained via plasma surface modification.^[^ ^215^ ^]^ The charged surface has the ability to enhance extracellular matrix (ECM) protein adhesion, inhibit TNF expression, and induce osteogenic differentiation of BMSCs through the surface charge mediated iNOS signaling pathway (**Figure** **14**a). Schröder et al.^[^ ^216^ ^]^ coated plasma polymers from allylamine (PPAAm) and acrylic acid (PPAAc) on a Ti surface. The presence of amine groups and carboxyl groups endowed the surface with positive and negative charges, respectively. The results indicate that a surface with a positive charge is more conducive to the adhesion and spread of human bone marrow mesenchymal stem cells (hMSCs). An internal electric field can also be built based on the pseudocapacitive effect of metal elements with variable valence. Wang et al.^[^ ^217^ ^]^ constructed C‐doped TiO_2_ nanotubes (TNT‐C) on biomedical Ti, which exhibited good captative properties. The captative film can be precharged using an external circuit before usage, and the charges can be well stored inside the film. When the charged TNT‐C film was in contact with the physiological environment, the high surface potential broke the respiratory chain of bacteria, resulting in a suitable antibacterial effect (Figure 14b).

Self‐activated implants can spontaneously form an electrochemical potential gradient around the implant surface. Therefore, self‐activated implants can adjust the transmembrane charge transfer without external power supply, which simplifies the system and increases biocompatibility. However, dynamic regulation cannot be achieved using self‐activated implants; that is, they cannot change the stimulus intensity according to real‐time demand. In addition, self‐activated implants are isolated systems that can hardly transmit any form of signals outside, and they do not have charge‐transfer monitoring abilities. Detecting the transmembrane charge transfer with self‐activated implants is a critical problem that is not yet solved. Recently, a family of resonator‐based sensors has been described that are wireless, battery‐less, and telemetry‐less and require no electrical connection.^[^ ^109^, ^218^ ^]^ The small sensors can be configured in various sizes to measure parameters including pH, mechanical force, and pressure. Although no charge‐transfer detecting capabilities of resonator‐based sensors have been reported, incorporating these sensors onto self‐activated implants is considered to be the most promising breakthrough point to endow self‐activated implants with charge‐transfer detecting abilities.

### 4.3 Stimuli‐Responsive Implants

**Figure 15:**
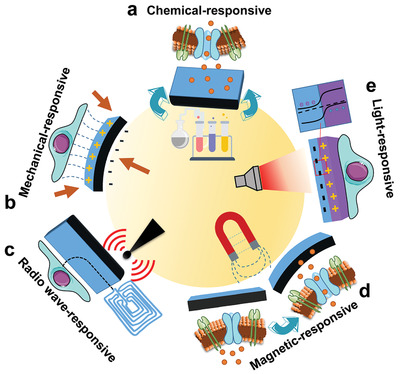
Strategies to induce charge transfer in a stimuli‐responsive way: a) chemical‐responsive system, b) mechanical‐responsive system, c) radio wave‐responsive system, d) magnetic‐responsive system, and e) light‐responsive system.

Based on the dynamic microenvironment in vivo, charge‐transfer regulation should be carried out in real‐time according to in situ demand. However, the above regulation methods based on ion concentration gradient and surface potential can only achieve static regulation of charge transfer. Therefore, regulating charge transfer in a dynamic manner has gained significant attention in this field.^[^ ^219^ ^]^ Various studies have obtained the in situ control of charge transfer via a stimuli‐responsive method. Different external and internal stimuli such as tissue chemical microenvironment (**Figure** **15**a), mechanical force (Figure 15b), radio waves (Figure 15c), magnetic field (Figure 15d), and light (Figure 15e) have been applied to induce charge transfer between the implant and the cells.^[^ ^19^, ^220^ ^]^ Some elaborately designed implant materials that were made of specific energy conversion materials can achieve the regulation of transmembrane charge transfer through converting the field energy into electric energy, which can further change the surface potential of the implant, the ion concentration around the implant, or affect the switching state of ion channels.

#### 4.3.1 Chemical‐Responsive Implants

After implantation, the chemical environments in the human body such as pH and degree of redox vary with time. For instance, in large bone defects, the microenvironment in the early stage is characterized by hypoxia and weak acidity, which gradually returns to normal in the later stage. Moreover, there are differences between the microenvironment of the normal and the diseased tissues. Notably, the tumor microenvironment is characterized by low pH and high reducibility compared with the normal tissues. Therefore, the chemical environment in vivo, specifically the redox properties and pH values, can be used as endogenous signals to regulate charge transfer.

**Figure 16:**
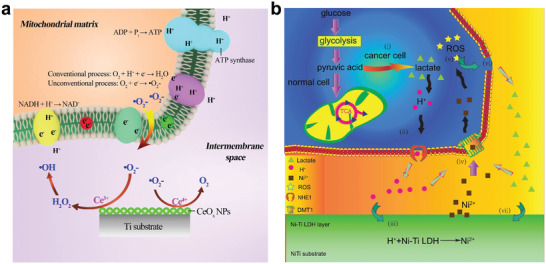
a) Illustration of the charge‐transfer process between CeO*_x_* NPs on titanium and the biological environment that was dependent on the redox level of the microenvironment, and the charge‐transfer process could modulate cell fate. b) Illustration of the selective anticancer mechanism of LDH films, the nickel ions transfer between LDH films and cells was determined by the pH of the microenvironment. a) Reproducedunder the terms of the Creative Commons CC‐BY license.^[^ ^221^ ^]^ Copyright 2018, TheAuthors, published by Wiley‐VCH. b) Reproduced with permission.^[^ ^224^ ^]^ Copyright 2015, American Chemical Society.

Loading redox‐sensitive transition metal elements to implants is an effective way of regulating charge transfer between the implant materials and the cells by taking advantage of their valence change under different redox conditions. Because of their valence‐changing ability, trace elements in the human body such as Fe, Ni, Mn, and Co are used in developing redox responsive coatings. Li et al.^[^ ^221^ ^]^ fabricated a cerium oxide coating on the surface of a Ti implant, which effectively consumed superoxide anions generated by abnormal cellular respiration via the change of valence state between trivalent cerium and tetravalent cerium, achieving real‐time regulation of the fate of the bone‐related cells (**Figure** **16**a). A similar effect can be achieved by loading other redox‐sensitive substances such as catechins and reducing polydopamine.^[^ ^222^ ^]^

The pH‐sensitive charge‐transfer regulating materials can realize the real‐time control of charge transfer by changing the concentration of the local ion around the cell in the environment with different pH values. The pH‐sensitive materials are divided into two types. The first type takes advantage of the differences in degradation rate in microenvironments with different pH values. Shen et al.^[^ ^223^ ^]^ fabricated an Mg/Zn MOF74 hybrid coating. Under acidic conditions of bacterial infection, the stability of the coating decreases, and Mg and Zn ions are released from the coating and then transferred to the bacteria, resulting in the death of bacteria. Under normal conditions, the material remains stable with less ion release, showing suitable biocompatibility. Similarly, we previously constructed a series of LDH films that are prone to degradation in the acidic microenvironment of the tumor tissue.^[^ ^224^ ^]^ The released Ni ions enter the cancer cells and kill them. The pH‐responsive ion transfer between the LDH film and cells endowed the constructed platform with selective anticancer effects (Figure 16b). The second type takes advantage of conformation changes in pH‐sensitive polymers. For instance, the conformation of poly(methacrylic acid) (PMAA) molecules can change with the variation in pH. They can expand under normal physiological conditions (pH 7.4), but contract at pH ≤ 6.0. When it is capped on the surface of the TiO_2_ NTs loaded with ions or drugs, the polymer function as a switch, which opens at low pH values and closes at high pH values. Thus, the pH‐responsive charge transfer can be realized.^[^ ^225^ ^]^ There are various polymers with similar pH‐responsive abilities, including *n*‐isopropyl acrylamide and ABA triblock copolymer.^[^ ^226^ ^]^

#### 4.3.2 Mechanical‐Responsive Implants

**Figure 17:**
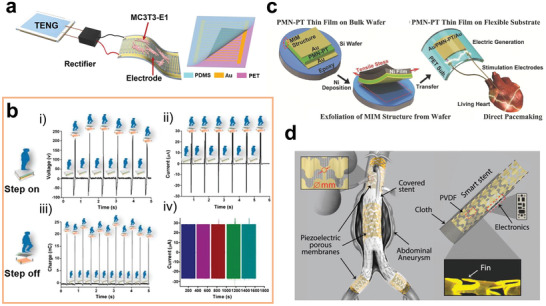
a) The schematic diagram of interdigitated electrode and the self‐powered electrical stimulator. TENG can work with human motions, and the typical b‐i) induced voltage, b‐ii) current, and b‐iii) transferred charge of TENG is driven by walking steps; b‐iv) stability of the TENG current output in 1500 s. c) Schematic illustration of the device fabrication process and stimulation test on a living heart. d) Example view of a smart stent that can monitor blood flow in the abdominal aneurysm. a) Reproduced with permission.^[^ ^228^ ^]^ Copyright 2019, Elsevier. b) Reproduced with permission.^[^ ^229^ ^]^ Copyright 2016, American Chemical Society. c) Reproduced with permission.^[^ ^231^ ^]^ Copyright 2014, Wiley‐VCH. d) Reproduced with permission.^[^ ^237^ ^]^ Copyright 2020, Institute of Electrical and Electronics Engineers Inc.

After the biomedical material is implanted in the human body, it faces a complex in vivo mechanical environment. The triboelectric/piezoelectric generator developed in recent years provides the possibility of utilizing the energy generated by mechanical friction in the human body directly.^[^ ^227^ ^]^ A triboelectric nanogenerator (TENG) is constructed based on the triboelectrification effect. When two different materials are in contact, their surfaces generate positive or negative electrostatic charges because of the electrical effect of the contact. When the two materials are separated by mechanical force, the positive and negative charges generated by electric contact are also separated. Such charge separation will generate a potential difference between the upper and lower electrodes. If a load is connected between the electrodes, the potential difference can drive electrons to flow between the electrodes through the external circuit. Tian et al.^[^ ^228^ ^]^ developed a TENG that can be implanted into the human body and connected it to an electrode (**Figure** **17**a). The TENG can generate energy from human movement and then convert it into electricity, regulating charge transfer without power supply. Additionally, the TENG can be connected to a scaffold directly to regulate charge transfer and cellular behavior. Guo et al.^[^ ^229^ ^]^ fabricated a scaffold with poly(3,4‐ethoxythiophene) (PEDOT)‐15% reduced GO (RGO) hybridized ultrafine fibers (80 m in diameter) and combined it with a highly effective TENG. Walking triggers the TENG to produce pulsed electrical analog signals (Figure 17b), which can regulate charge transfer around the scaffold and promote the neural differentiation of the bone marrow stem cells. A piezoelectric nanogenerator (PENG) converts mechanical energy into electrical energy via the piezoelectric effect. Piezoelectric materials are polarized after being stressed or compressed, and charges appear on their surfaces. In general, ferroelectrics are special cases of piezoelectric materials; therefore, the ferroelectric materials described in Section 4.2.2 can be used to develop the PENG. The maximum voltage and current output of the P(VDF‐TrFE) piezoelectric nanofiber was −1.7 V and 41.5 nA, respectively. The electrical signal induced by mechanical stimulation can effectively enhance the proliferation of preosteoblasts.^[^ ^230^ ^]^ Additionally, the PENG can be used as a power supplier for other bioelectrical devices. A flexible single‐crystalline Pb(Mg_1/3_Nb_2/3_)O_3_–*x*PbTiO_3_ PENG harvester achieved a self‐powered artificial cardiac pacemaker. The energy‐harvesting device converted tiny biomechanical motion into electric energy and generated a short‐circuit current of 0.223 mA and an open‐circuit voltage of 8.2 V, which is sufficient not only for meeting the standard for charging commercial batteries but also stimulating the heart without an external power source (Figure 17c).^[^ ^231^ ^]^

In addition to in vivo biomechanical motion, the mechanical force can be applied indirectly to regulate charge transfer. Ultrasonic wave is a promising strategy for applying mechanical stimulation and inducing in vivo charge transfer wirelessly. Ultrasound, recognized as an FDA‐approved technique, is one of the most promising strategies for clinical application.^[^ ^232^ ^]^ Ultrasonic waves have various advantages such as low energy loss, high penetration depth, and less harm to the human body.^[^ ^233^ ^]^ Currently, various ultrasonic transducers have been designed and applied to stimulate nerve, muscle, and bone tissue.^[^ ^19^, ^234^ ^]^ The core component of the ultrasonic receiver is made of piezoelectric ceramics. The receiver can convert the mechanical energy generated by ultrasonic waves into electrical energy through the piezoelectric effect to achieve the control of charge transfer. PZT is the most commonly used piezoelectric material because of its high electromechanical coupling coefficient. Most existing research has employed PZT to fabricate ultrasonic wave transducers.^[^ ^234^, ^235^ ^]^ Cochran et al.^[^ ^236^ ^]^ fabricated an ultrasonic transducer using PZT, achieving a current output of 1 mA, which was successfully applied to stimulate bone tissue healing. However, PZT contains Pb. Therefore, to minimize the safety concern, developing Pb‐free transducers has become the future trend. In a recent study, a piezoelectric PVDF membrane was fabricated with a blood stent, and it worked as the microwave receiving material simultaneously (Figure 17d). The smart stent can generate 0.23 mW of electrical power when exposed to ultrasonic stimulation, which can sufficiently operate low‐power wireless electronics.^[^ ^237^ ^]^ In another study, GO was added to PVDF via laser sintering to enhance its piezoelectric effect. The results indicated that the PVDF/0.3GO scaffold presented the best electrical performance, and the proliferation of cells cultured on this scaffold can be effectively enhanced when exposed to ultrasound.^[^ ^184b^ ^]^ To improve the biocompatibility of the transducer further, the device is encapsulated in Ti, PEEK, polydimethylsiloxane, and other materials with high biocompatibility.^[^ ^232^, ^238^ ^]^ Hinchet et al.^[^ ^232^ ^]^ encapsulated the transducer in polydimethylsilane, which effectively improved its histocompatibility. No excessive tissue response occurred after implanting the encapsulated device, and there was no significant change in animal behavior under ultrasound.

#### 4.3.3 Radio Wave‐Responsive Implants

**Figure 18:**
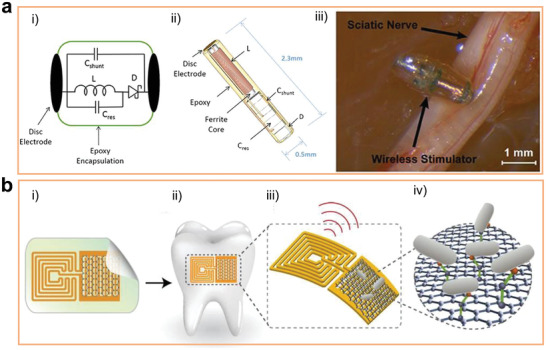
Illustration of the submillimeter, wireless stimulator: a‐i) The device consists of a coil (L) to receive inductive power, a capacitor (*C*_res_) for resonating the inductor, a Schottky diode (D) for rectification, and a shunt capacitor (*C*_shunt_) to facilitate rectification, a‐ii) assembly of the stimulator, and a‐iii) the electroparticle cathode was placed onto the sciatic nerve. b‐i) Graphene is printed onto bioresorbable silk and contacts are formed containing a wireless coil, b‐ii) biotransfer of the nanosensing architecture onto the surface of a tooth, b‐iii) magnified schematic of the sensing element, illustrating wireless readout, and b‐iv) binding of pathogenic bacteria by peptides self‐assembled on the graphene nanotransducer. a) Reproduced with permission.^[^ ^239^ ^]^ Copyright 2017, Frontiers Media S.A. b) Reproduced with permission.^[^ ^240^ ^]^ Copyright 2012, Nature Publishing Group.

Power can be transferred through radio waves wirelessly owing to the electromagnetic induction phenomenon. As a result, regulating charge transfer with the radio wave is widely used in wireless implant devices. Power transfer via radio waves requires antennas with feature sizes comparable to the radio wavelength. For commonly used submillimeter devices, their effective frequencies lie in the GHz range, where the radio radiation is absorbed by the body. Thus, the implant devices should be implanted near the surface of the skin to reduce safety concerns to stimulate implants in deep tissues. Therefore, near‐field inductive coupling (NIC) implant devices gain increasing attention. A conductive coil is connected to the implant as a power receiver, which delivers energy in the radio waves to regulate the surface potential of the implant, thus the transmembrane charge transfer process can be controlled accordingly. Freeman et al.^[^ ^239^ ^]^ designed a NIC implant for neural stimulating. In the implant, inductive coils with a diameter less than 1 mm received power from radio waves and charged the implant, creating a chemical potential gradient between the implant and neural tissue (**Figure** **18**a). Charges then flowed along the potential gradient, inducing tens of microamps of current, which stimulated the sciatic nerve in rats to produce a motor response. Mannoor et al.^[^ ^240^ ^]^ incorporated the conductive coil with a bacterial sensor and then pasted the device on the tooth enamel, achieving remote monitoring of respiration and bacterial detection in silva (Figure 18b). However, the NIC technique is sensitive to the angle and distance between the receiver and transmitter. Therefore, it is difficult to achieve a stable performance of the NIC in moving animals.^[^ ^241^ ^]^ Moreover, its application in implants deep inside the body is limited.

#### 4.3.4 Magnetic‐Responsive Implants

**Figure 19:**
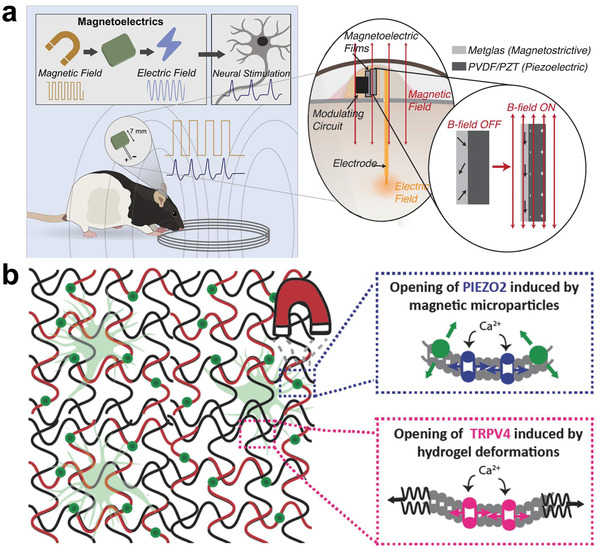
a) Diagram of a magnetic‐responsive device on a freely moving rat for wireless neural stimulation. Inset shows the operating principle whereby strain in the magnetostrictive layer is transferred to the dark gray piezoelectric layer, creating a voltage across the film. b) Mechanism of magnetomechanical stimulation of neurons with magnetic hyaluronic acid hydrogels. Mechanosensitive PIEZO2 channels are activated by magnetic microparticles embedded in the hydrogels through membrane stretching. On the other hand, mechanosensitive TRPV4 channels are activated by magnetic force‐induced hyaluronic acid hydrogel deformations. a) Reproduced with permission.^[^ ^242^ ^]^ Copyright 2020, Cell Press. b) Reproduced with permission.^[^ ^87^ ^]^ Copyright 2018, Wiley‐VCH.

The magnetic field is another energy source that can be applied wirelessly to regulate charge transfer. Compared with the radio or ultrasound waves, the magnetic field does not suffer from absorption or impedance mismatches at biological interfaces. Energy in the magnetic field can be transferred in vivo via two strategies. The first one uses inductive coils similar to those in NIC, which has been introduced in Section 4.3.3. The second strategy is to transform the magnetic field to electric stimulation using mechanical coupling magnetostrictive and the piezoelectric layers. The magnetic field generates strain in the magnetostrictive film as the magnetic dipoles align with the applied field. This strain exerts a mechanical force on the piezoelectric layer; consequently, voltage is generated to regulate the charge flow. Exploiting this strategy, Singer et al.^[^ ^242^ ^]^ bonded a rectangular magnetostrictive layer (Metglas) to a piezoelectric layer (PVDF or PZT). A significant increase in voltage across the film was detected after exposure to a magnetic field with a specific frequency. This magnetic stimulator was able to induce charge transfer in the deep brain and provide therapeutic deep brain stimulation in a moving rodent model for Parkinson's disease (**Figure** **19**a).

Additionally, the magnetic field can directly regulate charge transfer without transforming into an electrical field by loading magnetic nanoparticles (MNPs) in the implant. Adding MNPs to hydrogels is one of the most commonly used methods to construct magnetic responsive implants.^[^ ^243^ ^]^ Through loading the MNPs in hydroxypropyl methylcellulose, the obtained implants regulated the transfer of Ca ions between the material and the cells in a magnetic field, thus promoting cell proliferation.^[^ ^244^ ^]^ Filippi et al.^[^ ^245^ ^]^ loaded the MNPs into the human adipose tissue stromal vascular part (SVF) cells containing polyethylene glycol (PEG)‐based hydrogels. The composite can promote proliferation of the endothelial cell, calcification matrix deposition, and angiogenesis. In addition, magnetic hydrogel exhibits various physiological functions under the magnetic fields, such as tendon reparation, nerve stimulation, and osteogenesis reparation.^[^ ^246^ ^]^ Many researchers attributed the preferable charge‐transfer regulation ability of the MNP‐loaded implant to the deformation of magnetic hydrogel under the action of a magnetic force. The bending of the hydrogel exerts mechanical stimulus to the attached cells, affecting the off and on states of the mechanically sensitive ion channels located on the cell membrane (such as Piezol1, Piezo2, and TRPV4),^[^ ^87^, ^247^ ^]^ which realizes the regulation of charge transfer between the cells and the implants (Figure 19b).

#### 4.3.5 Light‐Responsive Implants

**Figure 20:**
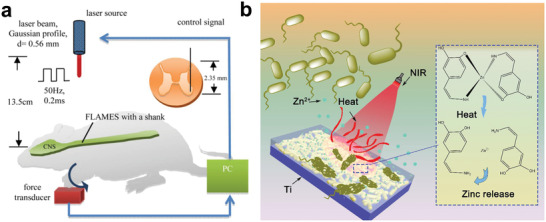
a) Schematic of the intraspinal cord microstimulation setup for in vivo testing of floating light‐activated microelectrical stimulator in rats. b) Illustration of the light‐triggered zinc ion transfer process between bacteria and the implant. a) Reproduced with permission.^[^ ^248^ ^]^ Copyright 2011, IOP Publishing Ltd. b) Reproduced with permission.^[^ ^255^ ^]^ Copyright 2020, The Royal Society of Chemistry.

Applying light irradiation to regulate charge transfer is based on two optical effects: photovoltaic and photothermal effects. Photovoltaic effect refers to the phenomenon in which a PN junction produces electrodynamic potential under light illumination. Based on this effect, Abdo et al.^[^ ^248^ ^]^ fabricated a light‐activated electrical stimulator with two cascaded GaAs photodiodes. Near‐infrared (NIR) pulses activated the device to induce potential alteration, which can result in charge transfer and stimulus current in vivo (**Figure** **20**a).^[^ ^248^ ^]^ Constructing a PN junction on the surface of the implant is a simpler way of making use of the photovoltaic effect to regulate charge transfer. Electrons and holes can be enriched on each side of the constructed junction after being illuminated, and a local electric field is built. Various novel semiconductors such as metal sulfide,^[^ ^249^ ^]^ metal–organic frameworks (MOFs),^[^ ^250^ ^]^ C_3_N_4_,^[^ ^251^ ^]^ and GO^[^ ^252^ ^]^ were used to construct PN junctions on the surface of the implant. When the constructed platform is exposed to light, it exhibits antibacterial, angiogenesis, or osteogenic effects. The preferable physiological function was attributed to the light‐induced electric field formed in the PN junction, which regulates the charge transfer between the cells and the implants.^[^ ^253^ ^]^

The photothermal effect is the phenomenon in which the temperature of the material increases after being irradiated by light. This is attributed to the interaction between the photon and lattice. The photon transfers its energy to the lattice, which intensifies the lattice vibration, thus resulting in the temperature increase. The photothermal effect produces hot electrons with high energy, which be transferred directly to the physiological environment to regulate the cell behavior. In addition, the increase in temperature promotes the ion release from the material, changing the ion concentration around the implant material and thus promotes the charge transfer between the implant and the cells.^[^ ^254^ ^]^ For instance, Yang et al.^[^ ^255^ ^]^ designed a composite film composed of Au nanorods and Zn‐containing polydopamine on the surface of Ti. The temperature of the coating increased under infrared irradiation, which effectively promoted the release of Zn ions from the coating (Figure 20b). The released Zn ions were then transferred into the bacteria to inhibit their growth. A similar strategy was used to promote the transfer of butyrate ions between the nitinol stent and cancer cells, producing a selective anticancer effect.^[^ ^256^ ^]^

By coupling with external fields such as light, magnetic fields, and microwaves, the stimuli‐responsive implants convert the energy of the external field into electrical energy; thus, regulating the electrochemical potential. Stimuli‐responsive implants are also wireless devices with various advantageous characteristics such as simplicity and convenience. In addition, they can overcome the defects of self‐activated implants to realize dynamic regulation and real‐time detection of charge transfer. However, the application of stimuli‐responsive implants is limited by the penetration depth of the external field to the human body. Nowadays, most stimuli‐responsive implants are only applicable to the stimulation or detection of charge transfer in the superficial tissues. Moreover, exposure to various external fields for a long time may cause damage to the human tissue, increasing the safety concerns of these implants. Therefore, developing new implant materials with high external field sensitivity will enhance regulation and detection precision, and decrease the required intensity of the external field; thus, reducing the possible human tissue damage.

## 5 Clinical Challenges of Implants with Charge‐Transfer Monitoring or Regulating Abilities

### 5.1 Biocompatibility and Foreign Body Reaction (FBR)

**Figure 21:**
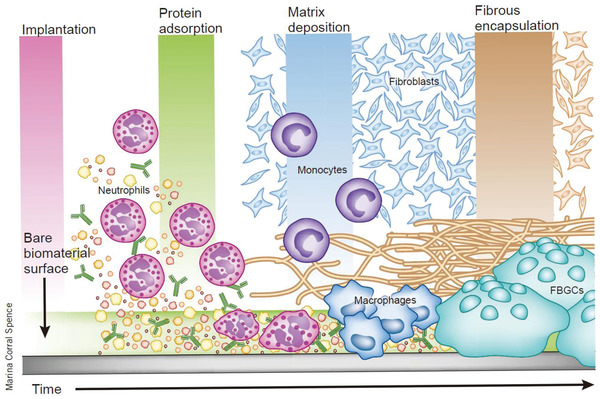
Schematic of the host response at different points of time upon implantation of a biomaterial. Reproduced with permission.^[^ ^260^ ^]^ Copyright 2013, Nature Publishing Group.

Biocompatibility is the first consideration in designing implants with charge transfer or regulating abilities, which is also the most important characteristic that distinguishes an implantable medical device from any other apparatus. Although biocompatibility is a term broadly used in biomaterials science, its actual meaning is still ambiguous. In 2008, Williams^[^ ^257^ ^]^ defined biocompatibility as follows: “Biocompatibility refers to the ability of a biomaterial to perform its desired function with respect to a medical therapy, without eliciting any undesirable local or systemic effects in the recipient or beneficiary of that therapy, but generating the most appropriate beneficial cellular or tissue response in that specific situation, and optimizing the clinically relevant performance of that therapy.” With regard to implant with charge‐transfer monitoring or regulating abilities, the most unfavorable factor that compromises biocompatibility is its rapid isolation from the body by an immune‐mediated FBR,^[^ ^258^ ^]^ which will inhibit the charge transfer between tissue and implant.^[^ ^259^ ^]^ As illustrated in **Figure** **21**,^[^ ^260^ ^]^ the FBR starts immediately after implantation, the biomedical device comes in contact with blood and adsorb serum proteins on its surface. Then the immune system deploys monocyte and macrophages to the implant, which can further fuse to form a multinucleated giant cell. The subsequent secretion of proangiogenic and fibroblast‐recruiting factors by activated immune cells results in the formation of a fibrous capsule around the biomaterials within 2–4 weeks.^[^ ^261^ ^]^ The fibrous layer has very high impedance,^[^ ^115^ ^]^ preventing the charge‐transfer process. It has been verified that even when the implant is made of nonreactive biomaterials, a fibrotic tissue around 100 µm in thick can build up.^[^ ^262^ ^]^ The FBR effects are deleterious to the function of the implant, especially to the device whose function relies on the charge‐transfer process between tissue and the implant. Besides, these unwanted effects cause significant discomfort and pain for the patient.^[^ ^262^, ^263^ ^]^ How to evade FBR has become the largest challenge in designing implants with charge‐transfer monitoring or regulating abilities.

### 5.2 Strategies to Evade the Foreign Body Reaction

#### 5.2.1 Physical Feature Optimization

Immune cells are able to sense the physical properties of biomaterials. The physical parameters such as size,^[^ ^264^ ^]^ shape,^[^ ^265^ ^]^ surface topography,^[^ ^266^ ^]^ porosity,^[^ ^267^ ^]^ and mechanical properties^[^ ^268^ ^]^ are demonstrated to affect protein adsorption and immune cell behaviors. Plenty of researchers are devoted to reducing FBR by changing the physical properties of implants.

##### Size and Shape

The phenomenon that implants’ geometry plays an important role in modulating FBR and fibrosis has been recognized in the 1970s. Matlaga et al.^[^ ^265^ ^]^ processed medical‐grade polymers into rods with circular‐, triangular‐ and pentagonal‐shaped cross‐sections, and evaluated the FBR was by implanting these materials into rat gluteal muscles for two weeks. Among the geometries evaluated, circular samples exhibited the least amount of FBR, followed by pentagonal and then triangular. Another research showed that the shape of percutaneous implants is a major factor influencing the polarization of macrophage, and found that a device with a smooth contour and no acute angles induces less FBR and is more biocompatible.^[^ ^269^ ^]^ Also, some studies focus on the effect of implant size on the immune response. Veiseh et al.^[^ ^264^ ^]^ constructed a series of implanted spheres with various diameters. They observed that spherical materials that are 1.5 mm in diameter or greater are more biocompatible than their smaller‐sized counterparts. This effect was verified to be independent of total implanted surface area and applicable across a broad spectrum of materials, including hydrogels, plastics, metals, and ceramics. Since these notable researches, it has long been a practical principle to design implant with smooth surface, which is likely to be more biocompatible than those with sharp edges, and chamfering are widely used in fabricating different kinds of implants.^[^ ^270^ ^]^

##### Roughness, Topography, and Porosity

The surface roughness and topography of the implant are known to affect macrophage attachment and phenotype and ultimately determining the FBR. In general, an implant with a smooth surface induces a lower inflammatory response,^[^ ^269^ ^]^ which has been a consensus of the design of implantable devices. However, plenty of recent researches indicated implants with nano or microsurface structures formed a thinner capsule than the smooth surface,^[^ ^266^, ^271^ ^]^ providing opportunities for the design of “immune‐instructive” topographies to modulate FBR. As an example, our group constructed uniform nanoleaf‐, nanosponge‐, and nanowire‐like structures on the surface of titanium.^[^ ^84^ ^]^ We found that the aspect ratio of nanostructures can regulate immune cell behavior, and nanostructures with lower aspect ratio exhibit thinner fibrous capsules. Therefore, no generalizable criterion of the relationship between surface texture and FBR exists. In order to investigate the relationship between topography and immune response, Vassey et al.^[^ ^266^ ^]^ designed a diverse library of 2176 micropatterns via an algorithm, and performed an unbiased screening of the topographical library with a machine learning algorithm, to identify topographies that promote both the attachment and polarization of macrophages. The researchers found that micropillars 5–10 µm in diameter play a dominant role in driving macrophage attachment compared to many other topographies screened, and it is a combination of pattern area and density of the micropillars that modulate the immune response. Surface porosity has also been identified as an important parameter affecting FBR. Generally, materials with larger pore sizes can reduce the inflammation response and FBR.^[^ ^267^, ^272^ ^]^ Sussman et al.^[^ ^267^ ^]^ compared the FBR to hydrogel scaffolds of different pore size, and found the scaffold with pore diameter of around 34 µm reduced fibrosis comparing to nonporous and 160 µm porous implant. In addition to porosity, scaffold orientation has been demonstrated to regulate the immune response. It was reported that the aligned orientation of scaffold fibers suffered from minimized FBR compared to randomly oriented fibers.^[^ ^273^ ^]^

As discussed in Section 4.1.1, alternating the surface topography is a widely used strategy to alleviate the FBR of an implant with charge transfer or regulating abilities.^[^ ^274^ ^]^ As an example, Tan et al.^[^ ^275^ ^]^ evaluated the surface texture on the behavior of microglia, which is an innate immunocyte in the central nervous system and responds to the gliosis process. Results showed that microglia proliferation was hampered by nanoporous samples. A similar conclusion was obtained by research conducted by Chapman et al,^[^ ^129b^ ^]^ who constructed a nanoporous gold (np‐Au) electrode via an alloy corrosion process. The nanotopography of the np‐Au reduced astrocyte surface coverage while maintaining high neuronal coverage, which is expected to alleviate FBR and prolong the in vivo working time of the electrode.

##### Mechanical Properties

Mismatch of mechanical properties between tissue and implant is also an influencing factor that trigger immune response.^[^ ^276^ ^]^ Mechanical‐mismatch‐induced FBR is especially significant to the implant for soft tissues. As discussed in Section 4.1, mismatch of mechanical properties between the neural implant and neural tissue leads to the generation of the glial sheath,^[^ ^115^ ^]^ which does harm to the charge‐transfer detection and regulation. The strategy to improve the mechanical and compatibility, and reduce FBR of implant involves implant geometry designing and materials engineering. The stiffness *D* of materials can be expressed as follows

(5) $$D\;=\frac{Eh^{3}}{12\left(1-v^{2}\right)}\;$$

where *h* is the thickness of the implant, *E* is its elastic modulus of the material, and *v* is its Poisson's ratio. Therefore, the bending stiffness scales linearly with the elastic modulus of the material and cubically with its thickness, so either reducing the dimension of the device or using low‐modulus materials to construct the implant device can reduce its stiffness and alleviate FBR. Keeping the above equation in mind, it is easy to understand why implantable devices with the shape of ultrathin film or thin fiber offer high compliance to soft tissues. A lot of implants with charge‐transfer regulating or detecting abilities are designed in the form of ultrathin film or fiber.^[^ ^14^, ^277^ ^]^ Likewise, researchers designed a neural probe that includes a fiber‐shaped electrode array with a diameter of 5 µm, rendering the bending stiffness much smaller than that of steel microwires. Benefiting from the conformal contacts on the target tissue, the constructed probe suffers less from FBR and can be reliably worked in freely moving mice by up to 2 months.^[^ ^278^ ^]^ It worth mentioning that the change of surface topography will also result in stiffness alteration. Therefore, the phenomenon that porous materials exhibited reduced FBR may stem from the decreased surface stiffness of porous implant compared to the nonporous counterparts, which reduces the mechanical strain between implant and tissue; thus, enhances the mechanical adaptation.

Based on Equation (5), wrapping or coating the implants with inherently low‐modulus elastomeric materials, such as hydrogels, is another method to reduce overall stiffness. Some representative examples have been discussed in Section 4.1.2. Benefiting from the intrinsic softness of the elastomeric materials, the coated or encapsulated implant can maintain a stable contact and charge transfer with the target tissue even under continued random deformation resulted from the surrounding environment.^[^ ^127^, ^279^ ^]^

#### 5.2.2 Chemical Modification

##### Anti‐Inflammatory Drug Loading

Loading anti‐inflammatory drugs or biomolecules is the most used approach to reduce the immune response to foreign objects.^[^ ^280^ ^]^ There are various kinds of drugs that can alleviate FBR, such as dexamethasone (DEX) and salicylic acid.^[^ ^258^ ^]^ DEX is the most used anti‐inflammatory drug, which is a synthetic glucocorticoid, it is able to diminish migration and activate immune cells, upregulate anti‐inflammatory cytokines, and reduce collagen production around the implant.^[^ ^281^ ^]^ Numerous works have incorporated DEX into coating deposited on the implant for local drug delivery. Zhong and Bellamkonda^[^ ^282^ ^]^ loaded DEX into a nitrocellulose coating on neural electrodes, and found the release of DEX reduced inflammation at 1 week after implantation, but the anti‐inflammatory effect disappeared 4 weeks postimplantation, possibly because the drug‐loaded amount was not high enough. In order to prolong the drug release term, researchers have screened various drug loading materials, most of which are organic materials such as liposomes,^[^ ^283^ ^]^ alginate hydrogel,^[^ ^284^ ^]^ nitrocellulose,^[^ ^282^ ^]^ poly(ethyl‐vinyl) acetate,^[^ ^285^ ^]^ and poly (lactic‐*co*‐glycolic acid).^[^ ^286^ ^]^ Inorganic drug loading layer, including titania,^[^ ^287^ ^]^ silica,^[^ ^288^ ^]^ and hydroxyapatite,^[^ ^289^ ^]^ is currently a new research focus, due to their higher biocompatibility. As a representative example, Li et al.^[^ ^288^ ^]^ grew vertical aligned mesoporous silica thin film alongside the walls of the titania nanotubes array. DEX can be effectively loaded into the hierarchical two‐layered nanotubular structure, and its release enhanced early adhesion of osteoblast. However, these nonconducting organic or inorganic layers may inhibit charge transfer between tissue and the implants, various conducting drug loading layers, such as graphene,^[^ ^290^ ^]^ carbon nanotube,^[^ ^291^ ^]^ and conducting polymers,^[^ ^292^ ^]^ were developed specifically for the charge‐transfer‐controlling implants. Kojabad et al.^[^ ^293^ ^]^ constructed conducting polypyrrole nanotube array on the neural microelectrodes and loaded DEX as dopant during the polymerization process. The drug loading coating can decrease charge‐transfer impedance, and in vitro experiments showed that DEX could release from the coating, effectively reducing the number of astrocytes, which indicated the drug loading coating can inhibit FBR without sacrificing the charge‐transfer detecting abilities of the neural electrode.

Growth factors and cytokines can regulate the macrophage phenotype and thus affect FBR. The continuous release of anti‐inflammatory cytokines such as interleukin‐4 (IL‐4) and interleukin‐10 (IL‐10) by implants is another approach to prevent FBR.^[^ ^294^ ^]^ However, it is difficult to maintain the activity and clinical required concentration of the cytokines for a long time. Therefore, co‐delivery of anti‐inflammatory drugs and cytokines appear to be a better choice. For example, the combined release of DEX and vascular endothelial growth factor (VEGF) has been demonstrated to minimize fibrosis and overcome the antiangiogenic effect of DEX.^[^ ^295^ ^]^

Although various anti‐inflammatory molecules and drugs are found to possess the ability to resist FBR, it should be noted that excessive immunosuppression is harmful to the body. In addition, stability, toxicity, and possible side effects should be considered. It has been reported that if extra amount of DEX releases, it can trigger serious side effect.^[^ ^296^ ^]^ Also, the implants still have to face immune reactions after the drugs or cytokines are completely released, so the drug loading strategy can hardly work for a long time. It remains a great challenge to design drug or biomolecule loading systems that achieve controlled and long‐term drug release.

##### Anti‐Inflammatory Layer Fabricating

Artificial materials inevitably trigger the immune response and encounter FBR, while natural biomaterials in the ECM are reported to be immunoprivileged, and may escape from FBR.^[^ ^297^ ^]^ Therefore, encapsulating the artificial implant with a layer of ECM‐derived biomaterials can be an effective approach to avoid immunological rejection. Numerous types of natural biomaterials in ECM, including gelatin, collagen, fibrin, and various kinds of polysaccharides (chondroitin sulfate, chitosan, hyaluronic acid, etc.), have been decorated on the surface of implants to resist immune response.^[^ ^298^ ^]^ For instance, Li et al.^[^ ^299^ ^]^ coated chondroitin sulfate on the surface of polyethylene terephthalate graft. The modified implant promoted macrophage polarizing to the M2 phenotype, switching the local immune microenvironment from pro‐inflammatory to anti‐inflammatory. Oakes et al.^[^ ^300^ ^]^ decorated a penetrating microelectrode array with ECM derived from astrocyte, and found that the ECM coating could reduce the FBR surrounding the electrode implanted in rat cortex, decrease the astrogliosis response 8 weeks after implantation. More examples can be found in the review written by Zhang et al.^[^ ^301^ ^]^

With the development of materials science and engineering, some kinds of synthetic materials with an intrinsic anti‐FBR property are designed, including zwitterionic materials,^[^ ^302^ ^]^ modified alginates,^[^ ^303^ ^]^ and polypeptide materials.^[^ ^304^ ^]^ The FBR is stemmed from nonspecific protein adsorption, so the major characteristic of anti‐FBR materials is their antiprotein adsorption abilities in complex in vivo environments. Constructing a layer of these antifouling materials on the surface of implants has become another effective strategy to resist FBR. Zwitterionic materials are the most investigated antifouling coating materials. They have equal anionic and cationic groups, rendering them highly hydrophilic, thus are promising to be used for antifouling, anticoagulant, and anti‐FBR.^[^ ^305^ ^]^ Golabchi et al.^[^ ^306^ ^]^ prepared an antifouling zwitterionic coating on the surface of the neural probe, and the probe was implanted in the mouse brain for 7 days. The coated probes presented reduced microglial activation, suggesting that the zwitterionic film could suppress the inflammatory around the implant. However, the mechanical property of zwitterionic hydrogel is poor, limiting its clinical application. Liu et al.^[^ ^307^ ^]^ designed a new class of zwitterionic hydrogels by introducing triazole moieties that could form energy‐dissipating pi–pi stacking. The triazole‐zwitterionic hydrogel presented much more mechanically robust than the conventional zwitterionic hydrogel, which could suffer from 250 tensile strain, 89% compressive strain, and 65% compression for at least 10 cycles without any crack. In addition, the anti‐FBR properties of the modified zwitterionic hydrogel were not compromised, which was verified by an in vivo subcutaneous implantation experiment.

Changing the physical parameter of implants is a promising strategy to modulate the immune response, which can provide a durable and resilient immune‐modulating signal. Investigations on reducing FBR via altering the physical properties of materials have provided scientists a lot of useful principles in designing implants, but there is no consensus rule that can be applied to all biomaterials. Besides, the FBR minimization effect of physical parameters is usually limited, which can hardly meet clinical needs. More importantly, there are clear requirements for the physical properties of the implant in specific applications. Loading anti‐inflammatory drugs is a more universal and flexible approach to dampen the immune response, but this strategy is relatively short‐lived and may induce side effects. Decorating the implant with antifouling materials seems to be a more promising approach to evade FBR, and suitable for different types of implants. Despite researchers have found numerous materials possessed good antifouling abilities, the materials with sufficient anti‐FBR abilities are rare. Up to now, only zwitterionic materials, modified alginates, and polypeptide materials show potential in alleviating immune response. However, most of the anti‐FBR materials are poor in mechanical properties, which limit their clinical application. Therefore, modifying the existing anti‐FBR materials to enhance their mechanical durability, exploring new anti‐FBR materials will be promising research fields.

## 6 Conclusions and Outlooks

Biomedical implants have developed from bioinert materials such as gold, polymethyl methacrylate, and zirconia, which only provide a “suitable combination of physical properties to match those of the replaced tissue,”^[^ ^308^ ^]^ toward smart implant devices that can provide not only therapeutic benefits but also diagnostic capabilities.^[^ ^4^, ^109^ ^]^ In recent years, scientists have designed numerous advanced biomedical implants, which have the ability to release or absorb bioactive ions, or impose electric stimuli to exert antibacterial, antitumor, osteogenesis, vascularization, wound healing, and neural stimulation biological function. All the above therapeutic benefits are verified to be derived from the charge‐transfer process between the implants and the cells. In addition, detecting the charge transfer is also a feasible method to record the physiological change in vivo for disease diagnose. Therefore, endowing biomedical implant devices with charge‐transfer monitoring or regulating abilities is a valuable domain in smart implant designing. In this review, we elaborated on advanced strategies, materials, and mechanisms to construct charge‐transfer‐controlling implants. We have summarized three types of charge‐transfer‐controlling systems, including wired, self‐activated, and stimuli‐responsive implants, which can sense the microenvironment in the human body or provide real‐time stimuli to achieve specific physiological function. However, it is still a challenging task to regulate or detect charger transfer in the conductive physiological medium with significant crosstalk. Several tradeoffs exist between achieving both charge‐transfer modulating and sensing abilities, increasing precision and sensitivity, simplifying systems, and biocompatibility, which are examined in detail below. 1) Wired implant can dynamically monitor and regulate the transmembrane charge transfer process with high precision. However, an external power supply is usually required. Besides, their mismatched mechanical and biological properties may result in fiber wrapping, which would reduce their sensitivity and working life. The research emphasis in the future is suggested to develop wireless chargeable batteries with small size and high biocompatibility and design nanosized or flexible electrodes with good biocompatibility, high charge injection ability, and good mechanical property matching with the human tissues. 2) Self‐activated implants can spontaneously build an electrochemical potential gradient around the implant surface to adjust the transmembrane charge transfer of cells without an external power supply. However, they cannot change the stimulus intensity according to real‐time demand, and most of them lack charge‐transfer monitoring ability because they cannot transmit signals to the outside. The research emphasis in the future is suggested to incorporate some sensors into self‐activated implants to endow them with charge‐transfer monitoring ability. 3) Stimuli‐responsive implants are kinds of wireless devices, which can convert the energy of the external field into electrical energy to regulate the electrochemical potential. They can realize the dynamic regulation and real‐time detection of transmembrane charge transfer. However, the application of stimuli‐responsive implants is limited by the penetration depth of the external field to the human body. The research emphasis in the future is suggested to develop novel materials with high external field sensitivity to enhance the regulation and detection precision and decrease the required intensity of the external field.

The major challenge in clinical application of the above three types of implants lies in the inevitable FBR, which leads to fibrosis capsules around the implants, inhibiting their communication with host tissues. Despite that several strategies including optimizing the physical parameters of the devices, loading anti‐inflammatory drugs, and constructing anti‐inflammatory coatings have been proposed to evade FBR, alleviating the FBR to implants with charge‐transfer regulating of recording abilities is still a challenging task. Exploration of new anti‐FBR materials which possess the property of high electronic or ionic conductivity is an emerging field, and we believe that these new types of anti‐FBR coatings will found application in implants with charge‐transfer‐controlling abilities in the near future.

The direction and flux of the transmembrane flowing ions or electrons are jointly affected by the electrochemical potential gradient of the charged particles and the activation state of the charge‐transfer‐related proteins. However, the charge‐transfer detection or regulation of the above implants is mostly achieved through sensing or adjusting the electrochemical potential of the transported charges but not activating the charge‐transfer channels. The regulation of chemical potential is a macroscopic method, which is not sensitive to the type of the charged particles. But the cellular behavior is highly related to the types of the charged particles passing through the cell membrane. Implantable devices that monitor or regulate the activation states of charge‐transfer channels enable detecting or modulating the transmembrane transfer of a specific charged particle, benefiting from the charge‐type specificity of the charge‐transfer‐related membrane proteins. Unfortunately, the factors activating the charge‐transfer channels are complex and still unclear. Further understanding the fundamental mechanisms underlying the activation of charge‐transfer‐related channels can provide new insights into the design of a smarter implant that monitors and regulates charge transfer with higher accuracy.

## Conflict of Interest

The authors declare no conflict of interest.
